# Modeling human somite development and fibrodysplasia ossificans progressiva with induced pluripotent stem cells

**DOI:** 10.1242/dev.165431

**Published:** 2018-08-23

**Authors:** Taiki Nakajima, Mitsuaki Shibata, Megumi Nishio, Sanae Nagata, Cantas Alev, Hidetoshi Sakurai, Junya Toguchida, Makoto Ikeya

**Affiliations:** 1 Department of Clinical Application, Center for iPS Cell Research and Application, Kyoto University, Kyoto 606-8507, Japan; 2 Department of Tissue Regeneration, Institute for Frontier Life and Medical Sciences, Kyoto University, Kyoto 606-8507, Japan; 3 Department of Cell Growth and Differentiation, Center for iPS Cell Research and Application, Kyoto University, Kyoto 606-8507, Japan; 4 Department of Orthopaedic Surgery, Graduate School of Medicine, Kyoto University, Kyoto 606-8507, Japan

**Keywords:** Differentiation, Disease modeling, Fibrodysplasia ossificans progressiva, Induced pluripotent stem cells, Paraxial mesoderm

## Abstract

Somites (SMs) comprise a transient stem cell population that gives rise to multiple cell types, including dermatome (D), myotome (MYO), sclerotome (SCL) and syndetome (SYN) cells. Although several groups have reported induction protocols for MYO and SCL from pluripotent stem cells, no studies have demonstrated the induction of SYN and D from SMs. Here, we report systematic induction of these cells from human induced pluripotent stem cells (iPSCs) under chemically defined conditions. We also successfully induced cells with differentiation capacities similar to those of multipotent mesenchymal stromal cells (MSC-like cells) from SMs. To evaluate the usefulness of these protocols, we conducted disease modeling of fibrodysplasia ossificans progressiva (FOP), an inherited disease that is characterized by heterotopic endochondral ossification in soft tissues after birth. Importantly, FOP-iPSC-derived MSC-like cells showed enhanced chondrogenesis, whereas FOP-iPSC-derived SCL did not, possibly recapitulating normal embryonic skeletogenesis in FOP and cell-type specificity of FOP phenotypes. These results demonstrate the usefulness of multipotent SMs for disease modeling and future cell-based therapies.

## Abstract (precis)

**Summary:** Protocols for the differentiation of human iPSCs to somite derivatives (myotome, sclerotome, syndetome and dermatome) are developed and applied to the modeling of the bone disease fibrodysplasia ossificans progressiva.

## INTRODUCTION

Recapitulation of endogenous signaling environments is considered to be key for the induction of desired cell types from pluripotent stem cells (PSCs). Developmental biology studies have shown that activin/nodal/transforming growth factor β (TGFβ) signaling induces mesendoderm formation and bone morphogenetic protein (BMP) signaling induces mesoderm to form from PSCs, whereas blockade of these signals induces the development of neural cells (Bernardo et al., 2011; Sumi et al., 2008; Chambers et al., 2009; Fasano et al., 2010). Notably, mesoderm formation induced by activin/nodal/TGFβ and BMP signaling results mainly in the production of lateral plate mesoderm (LPM), a lateral (ventral) subpopulation of mesoderm, but not of paraxial mesoderm, a mesoderm subpopulation formed between the neural tube and LPM. Although several attempts have been performed to induce paraxial mesoderm by modifying activin/nodal/TGFβ-based protocols, the induction rate remains relatively low (approximately 20%) (Sakurai et al., 2012).

Recently, several groups reported successful induction of paraxial mesoderm based on different approaches (Chal et al., 2015, 2016; Umeda et al., 2012; Loh et al., 2016; Xi et al., 2017). In these studies, cells were cultured with or without activin/nodal/TGFβ signaling inhibitors, which induce neural (dorsal) fate (Chambers et al., 2009; Fasano et al., 2010), and with relatively high concentrations of glycogen synthase kinase 3 (GSK3) inhibitors (WNT signaling activators). Using these protocols, the induction rate of paraxial mesoderm reached 70-95% (Loh et al., 2016; Xi et al., 2017). This conversion from neural to paraxial mesoderm suggests a common precursor of the neural and paraxial mesoderm during embryogenesis, known as neuromesoderm progenitors or axial stem cells (Takemoto et al., 2011; Tzouanacou et al., 2009; Gouti et al., 2017). This prediction was further supported by the observation that *Wnt3a* knockout mice show an ectopic (secondary) neural tube rather than loss of paraxial mesoderm (Takada et al., 1994; Yoshikawa et al., 1997).

Despite progress in the induction of paraxial mesoderm and its derivatives, several limitations remain. During vertebrate development, paraxial mesoderm first forms the presomitic mesoderm (PSM) posteriorly and somites (SMs) anteriorly (Tam and Beddington, 1987; Aulehla and Pourquié, 2010). SMs eventually differentiate into the dermomyotome (DM) dorsally and sclerotome (SCL) ventrally (Christ and Scaal, 2008). The DM gives rise to the dermatome (D), a precursor of the dermis, and to myotome (MYO), a precursor of the skeletal muscle; additionally, a subpopulation of SCL forms the syndetome (SYN), a precursor of tendons and ligaments (Brent et al., 2003). To demonstrate the full competence of SMs induced from PSCs, it is important to determine the multi-differentiation capacity of induced SMs into D, MYO, SCL and SYN. Although previous studies induced MYO and SCL, induction protocols for D and SYN have not been established. Moreover, although the LPM is a major source of mesenchymal stromal cells (MSCs) (Sheng, 2015), SMs might also be a source. However, no studies have induced MSC-like cells from PSCs through the paraxial mesoderm.

Here, we report the induction of SM derivatives including D, SYN and MSC-like cells. We also applied our protocols to a model of an intractable rare disease, fibrodysplasia ossificans progressiva (FOP), which is characterized by endochondral heterotopic ossification in soft tissues, and successfully recapitulated the disease phenotypes *in vitro*. Taken together, our comprehensive protocols are a powerful tool for modeling normal and abnormal human SM development.

## RESULTS

### Induction of PSM from human iPSCs

**Fig. 1.**
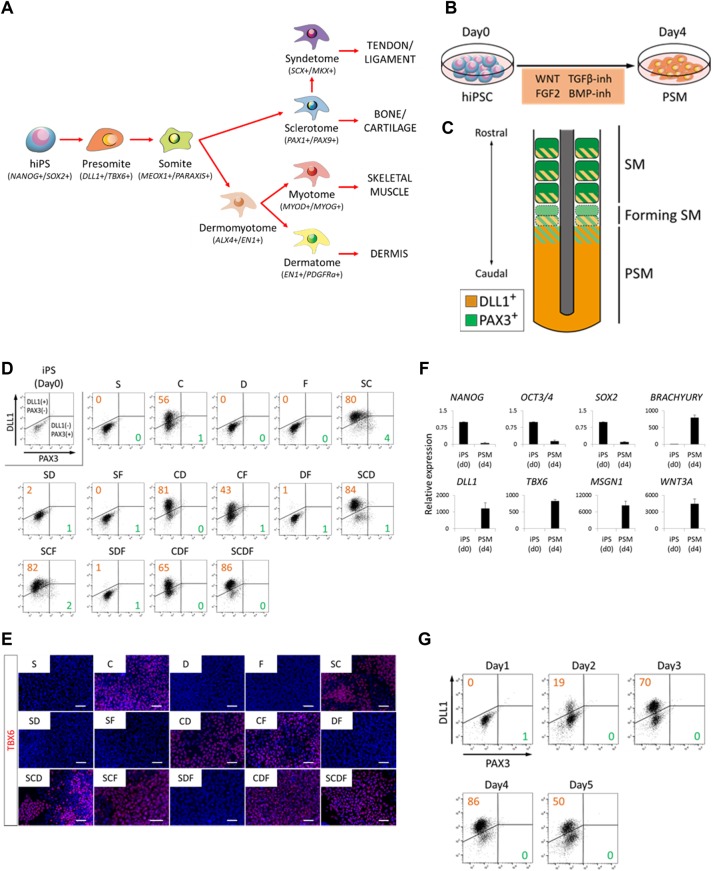
**Directed differentiation of** **human** **iPSCs toward PSM fate by combined WNT/FGF activation and TGFβ/BMP inhibition.** (A) Schematic view of hierarchical induction of SM derivatives. (B) Schematic view of a protocol for PSM induction from human iPSCs (hiPSCs) with WNT activator (CHIR99021, 10 μM), FGF2 (20 ng/ml), TGFβ inhibitor (SB431542, 10 μM) and BMP inhibitor (DMH1, 2 μM). (C) Expression pattern of *DLL1* and *PAX3* during somitogenesis. (D,E) Investigation of an optimized protocol for PSM induction assessed by FACS with anti-DLL1 antibody and PAX3-GFP (D) and immunocytochemistry analysis (E). Data were obtained from three biological replicates and representative data are shown. Images were acquired in representative areas of each condition (E). Cells were stained with anti-TBX6 antibody (red) and co-stained with DAPI (blue). The SCDF condition (combination of SB431542, CHIR99021, DMH1 and FGF2) most efficiently induced DLL1^+^ PSM among the 15 conditions considered based on previous developmental biology studies. (F) RT-qPCR analysis of markers for PSC and PSM at day 0 (d0) and day 4 (d4) of PSM induction. Gene expression of iPSCs and DLL1 sorted cells is shown. Error bars represent s.e.m. (*n*=3). (G) FACS with anti-DLL1 antibody to determine the optimum day for PSM induction. Scale bars: 50 μm. C, CHIR99021 10 μM; D, DMH1 2 μM; F, FGF2 20 ng/ml; S, SB431542 10 μM.

Human PSCs are considered equivalent to epiblast cells based on their cell morphology and signal requirements (Thomson et al., 1998). In the epiblast stage, the presumptive paraxial mesoderm is induced in the epiblast near the anterior primitive streak, which is located just posterior to the node (Iimura et al., 2007). These cells invaginate through the primitive streak, form the PSM, subsequently form SM cells through mesenchymal-to-epithelial transition, and eventually differentiate into the four SM derivatives (Fig. 1A) (Bénazéraf and Pourquié, 2013; Brent and Tabin, 2002).

To develop stepwise induction protocols from human PSCs to SM derivatives, we first examined the signaling environment of the presumptive paraxial mesoderm in the epiblast and early gastrulation stage of chick and mouse embryos. The primitive streak expresses *Wnt3*, *Fgf4* and *Fgf8* (Chapman et al., 2004; Hardy et al., 2011; Chapman et al., 2002). Additionally, based on observations in Wnt-reporter mice, canonical Wnt signaling may be activated in the primitive streak and PSM (Maretto et al., 2003). Follistatin, an extracellular inhibitor of activin/nodal/TGFβ, is expressed in the early paraxial mesoderm (Chapman et al., 2002). Chordin and noggin, extracellular inhibitors of BMP, are expressed in the node (Patwardhan et al., 2004; Streit and Stern, 1999; Chapman et al., 2002) and phosphorylated Smad1 is not detected in the presumptive PSM region (Faure et al., 2002), suggesting the suppression of BMP signaling in the presumptive PSM. Taken together, we predicted that the signaling environment of the presumptive/early paraxial mesoderm should be TGFβ-OFF/WNT-ON/BMP-OFF/FGF-ON. Moreover, to minimize the effect of growth factors secreted from feeder cells contained in the culture medium, human induced pluripotent stem cells (iPSCs) were first cultured under feeder-free conditions with mTeSR1 medium and growth factor-reduced Matrigel for 3 days, and then cultured in chemically defined medium (CDM) containing 10 μM SB431542 (activin/nodal/TGFβ inhibitor), 10 μM CHIR99021 (GSK3β inhibitor), 2 μM DMH1 (BMP inhibitor) and 20 ng/ml fibroblast growth factor 2 (FGF2) for 4 days (Fig. 1B). To detect the induction efficiency of PSM cells by fluorescence-activated cell sorting (FACS), the cell population positive for DLL1, a surface marker for PSM and the posterior portion of SMs, and negative for PAX3, a marker for newly formed and segmented SMs, was determined (Fig. 1C). Because PAX3 is a transcription factor, PAX3-GFP knock-in iPSCs (PAX3-GFP iPSCs) were used to track PAX3-positive cells during differentiation.

First, the effects of SB431542, CHIR99021, DMH1 and FGF2 were analyzed individually. Consistent with previous reports (Loh et al., 2016; Xi et al., 2017; Chal et al., 2015; Umeda et al., 2012; Sudheer et al., 2016), CHIR99021 efficiently induced DLL1^+^/PAX3-GFP^−^ cells (56.3±3.1%) (C in Fig. 1D and Fig. S1). The combination of CHIR99021 with SB431542 or DMH1 (SC and CD, respectively, in Fig. 1D) was found to induce DLL1^+^/PAX3-GFP^−^ cells efficiently (80.5±1.7% and 80.6±1.2%, respectively) (Fig. S1). As expected, SB431542 and CHIR99021 in combination with DMH1 (SCD) induced DLL1^+^/PAX3-GFP^−^ cells at high levels (83.8±1.1%), but DLL1^−^/PAX3-GFP^+^ cells also appeared, suggesting that the cells differentiated into PAX3^+^ SMs and/or neural cells under this condition. Therefore, we added all four molecules. Under this condition, DLL1^+^/PAX3-GFP^−^ cells were induced from PAX3-GFP iPSCs without any PAX3-GFP^+^ cells (85.4±0.4%) (SCDF in Fig. 1D and Fig. S1). Although there were no obvious differences among SCD, SCF and SCDF conditions in terms of the induction efficiency of DLL1^+^ cells (Fig. S1), SCDF accurately recapitulated the endogenous signaling environment. Therefore, we employed the SCDF condition for further analyses. The robustness of the protocol was demonstrated by the high induction efficiency of DLL1^+^ cells from other iPSC clones (201B7, 409B2, 414C2 and TIG118-4f) (Fig. S2). Successful induction of PSM under the SCDF condition was confirmed by immunocytochemistry with anti-TBX6, anti-brachyury (TBXT), and anti-CDX2 antibodies (Fig. 1E and Fig. S3) and by RT-qPCR of the PSM markers brachyury, *DLL1*, *TBX6*, *MSGN1* and *WNT3A* and the pluripotent markers *NANOG*, *OCT3/4* (*POU5F1*) and *SOX2* (Fig. 1F). We also confirmed that under the SCDF condition, the induction efficiency of DLL1^+^/PAX3-GFP^−^ cells peaked on day 4 (Fig. 1G and Fig. S4).

### Induction of SMs from PSM

**Fig. 2.**
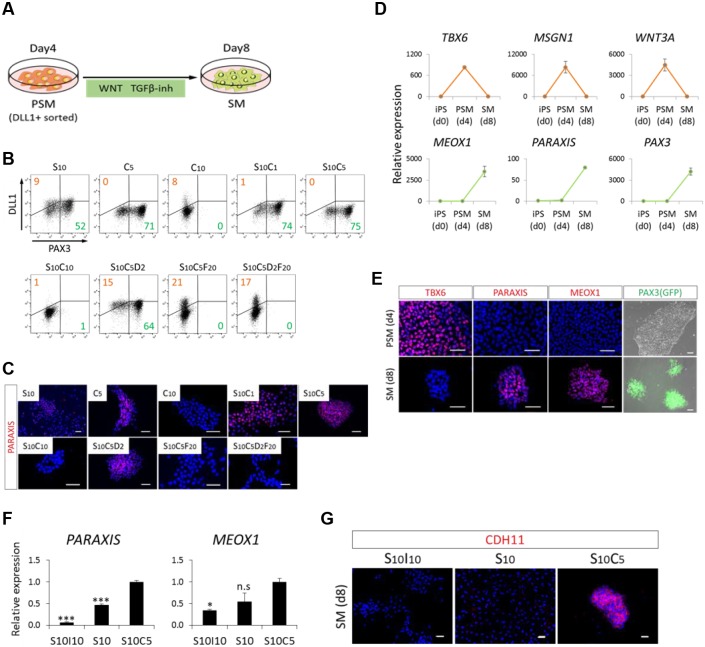
**Directed differentiation of PSM toward SM fate by combined WNT activation and TGFβ inhibition.** (A) Schematic view of a protocol for SM induction. Sorted DLL1^+^ PSM cells were treated with WNT activator (CHIR99021, 5 μM) and TGFβ inhibitor (SB431542, 10 μM) for 4 days. (B,C) Investigation of an optimized protocol for SM induction assessed by FACS with anti-DLL1 antibody and PAX3-GFP (B) and immunocytochemistry analysis (C). Cells were stained with anti-paraxis antibody (red) and co-stained with DAPI (blue). The SC condition [combination of WNT activator (CHIR99021, 5 μM) and TGFβ inhibitor (SB431542, 10 μM)] most efficiently induced PAX3-GFP^+^ SM among the nine conditions considered based on previous developmental biology studies. (D) RT-qPCR analysis of markers for PSM and SM at day 0 (d0), day 4 (d4) and day 8 (d8) from iPSCs. (E) Immunocytochemistry analysis and PAX3-GFP fluorescence at day 4 and day 8 (d8) from iPSCs. Cells were stained with TBX6, paraxis and MEOX1 antibodies (red) and co-stained with DAPI (blue) or PAX3-GFP fluorescence (green) was detected. (F,G) Effect of Wnt signaling on the quality of induced SMs. The characteristics of SM were assessed by RT-qPCR (F) and immunocytochemistry (G) analyses. Cells were stained with anti-CDH11 antibody (red). Error bars represent s.e.m. (*n*=3). *P<0.05; ***P<0.001 by Dunnett's multiple comparisons *t*-test compared with S10C5 (F) (n.s, no significant difference). Scale bars: 50 μm. C1, CHIR99021 1 μM; C5, CHIR99021 5 μM; C10, CHIR99021 10 μM; D2, DMH1 2 μM; F20, FGF2 20 ng/ml; I10, IWR1 10 μM; S10, SB431542 10 μM.

Next, we evaluated the induction of SMs from DLL1^+^ PSM cells sorted by FACS (Fig. 2A). The expression of PAX3-GFP was used as an SM marker. Because phosphorylated Smad2 is not observed in epithelial SMs (Faure et al., 2002) and because Wnt-reporter is active in SMs (Moriyama et al., 2007; Maretto et al., 2003), SB431542 and CHIR99021 were administered. After 4 days of induction, either 10 μM SB431542 or 5 µM CHIR99021 efficiently induced PAX3-GFP^+^ cells (52.1±0.8% and 70.7±0.1%, respectively); treatment with both compounds maximized induction (74.7±0.5%) (Fig. 2B and Fig. S5). However, higher levels of CHIR99021 (10 µM) failed to induce PAX3-GFP^+^ cells (0.3±0.0% in C10, and 0.7±0.1% in S10C10) (Fig. S5), suggesting that excess WNT signaling suppresses the induction of SMs. These results were confirmed by immunocytochemistry with an anti-paraxis (TCF15) antibody (Fig. 2C). The successful transition from PSMs to SMs was demonstrated by RT-qPCR analysis of several PSM markers (*TBX6*, *MSGN1* and *WNT3A*) and somite markers (*MEOX1*, paraxis and *PAX3*) (Fig. 2D). Immunocytochemistry with anti-TBX6, anti-paraxis and anti-MEOX1 antibodies and PAX3-GFP expression showed similar results (Fig. 2E).

To gain a more detailed understanding of the role of WNT signaling during SM induction, PSMs were cultured in SB431542 alone, or in SB431542 with CHIR99021 or with IWR1, an inhibitor of WNT signaling. As a result, paraxis and *MEOX1* expression were highly induced by CHIR99021 (Fig. 2F); interestingly, CDH11, a marker of epithelial SMs, accumulated at the cell-cell junction only following addition of SB431542 with CHIR99021 (S10C5 in Fig. 2G), suggesting the involvement of WNT signaling in SM epithelialization.

Because of the low survival rate of PAX3^+^ cells after FACS sorting, the cells described below were processed without sorting.

### Induction of MYO and D through DM

**Fig. 3.**
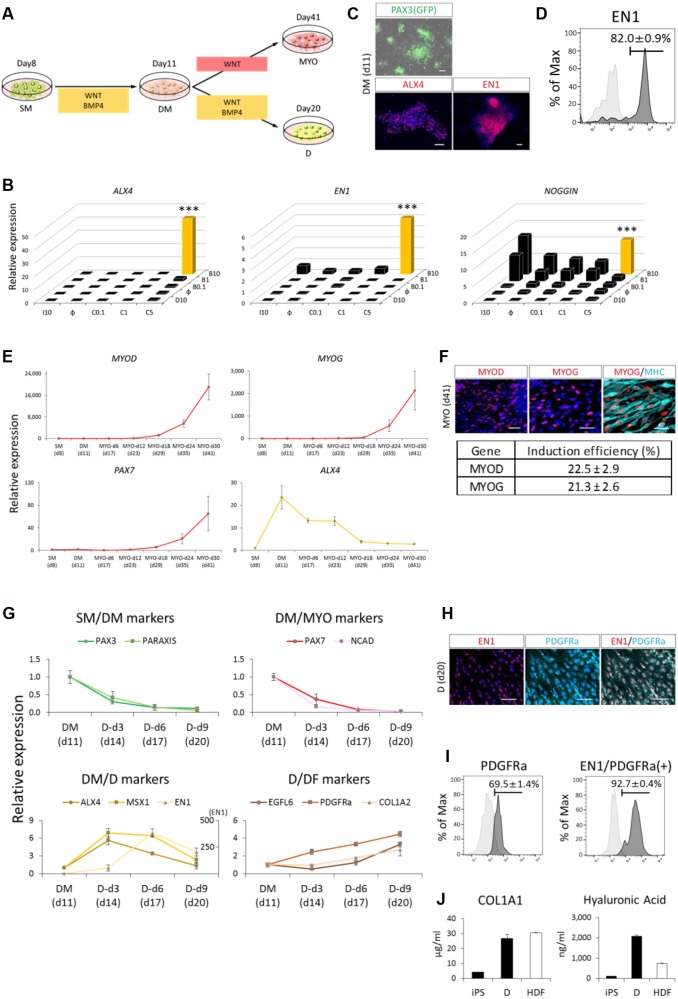
**Directed differentiation of SM toward MYO and D fate through DM.** (A) Schematic view of protocols for the induction of DM and its derivatives (MYO and D). For DM induction, induced SM was treated with WNT activator (CHIR99021, 5 μM) and BMP4 (10 ng/ml) for 3 days. For MYO induction, induced DM was treated with WNT activator (CHIR99021, 5 μM) for 30 days. For D induction, induced DM was treated with WNT activator (CHIR99021, 5 μM) and BMP4 (10 ng/ml) for 9 days. (B) Investigation of an optimized protocol for DM induction assessed by RT-qPCR at day 3. WNT and BMP signals were considered to be involved in DM induction based on previous developmental biology studies. *x*-axis: C0.1, C1, C5, CHIR 0.1, 1 and 5 μM, respectively; I10, IWR1 10 μM; ɸ, no compound (IWR1 or CHIR). *z*-axis: B0.1, B1, B10, BMP4 0.1, 1 and 10 ng/ml, respectively; D10, DMH1 10 μM; ɸ, no compound (DMH1 or BMP4). (C,D) Differentiation toward DM fate was assessed by PAX3-GFP fluorescence and immunocytochemistry (C), and FACS analysis of EN1^+^ cells (D). The mean±s.e.m. from three sets of experiments are shown. iPSCs were used as controls. (E,F) Differentiation toward MYO fate was assessed by RT-qPCR (E) and immunocytochemistry (F). (G) Differentiation toward D fate was assessed by RT-qPCR. The second vertical axis in DM/D markers indicates EN1 expression. The expression level of DM was set to one. In E and G, the number of days from iPSCs are indicated in brackets whereas the number of days of MYO or D induction are indicated after the cell type and hyphen (e.g. MYO-d6 indicates day 6 of MYO induction). (H,I) Differentiation was also assessed by immunocytochemistry (H) and FACS (I). The mean±s.e.m. from three sets of experiments are shown. DM was used as a control population (I). (J) Additionally, the amounts of collagen type I and hyaluronic acid protein in the culture medium were assessed by enzyme-linked immunosorbent assay. Scale bars: 50 μm. Error bars represent s.e.m. (*n*=3). ***P<0.001 by Dunnett's multiple comparisons *t*-test compared with no additional supplement control (ɸ,ɸ) (B).

Based on transplantation experiments using chick-quail embryos, SMs have been shown to adopt their fate according to the surrounding environment (Fomenou et al., 2005). The dorsal SM differentiates into the DM and subsequently into the MYO and D (Fig. 3A), whereas the ventral SM differentiates into the SCL. Wnt-reporter mouse embryos have been shown to exhibit activated Wnt signaling in the medial part of the dorsal SM (Moriyama et al., 2007; Maretto et al., 2003), whereas phosphorylated Smad1 staining indicated activation of BMP signaling in the lateral part of the SM (Faure et al., 2002). We therefore focused on WNT and BMP signaling in DM induction. Different concentrations of CHIR99021 (0, 0.1, 1 and 5 µM) were used to activate WNT signaling, and 10 µM of IWR1 was used to inhibit this signaling. Similarly, different concentrations of BMP4 (0, 0.1, 1 and 10 ng/ml) and 10 µM DMH1 were used to control BMP activity. *ALX4*, *EN1* and noggin were used as markers for the DM, and after several trials we found that the WNT-high and BMP-high condition efficiently induced these DM markers (Fig. 3B). This result was confirmed by immunocytochemistry with anti-ALX4 and anti-EN1 antibodies, PAX3-GFP, which is expressed in both the SM and DM (Fig. 3C and Fig. S6), and FACS using an anti-EN1 antibody (Fig. 3D).

To determine the differentiation capacity of the induced DM, we next attempted to induce MYO directly from the DM. To simplify this induction, we used CDM as the base medium. *Wnt1* and *Wnt3a* have been shown to play an essential role in inducing MYO in mice and chicks (Ikeya and Takada, 1998; Tajbakhsh et al., 1998; Galli et al., 2004), and the Wnt-reporter is activated in the dorsal-medial portion of the SM (Moriyama et al., 2007; Maretto et al., 2003). Additionally, follistatin and noggin, which are extracellular inhibitors of BMP, are expressed in the dorsal-medial lip (Hirsinger et al., 1997; Nimmagadda et al., 2005). Thus, we assessed simple administration of CHIR99021 in the CDM; we found that several myogenic markers, including *MYOD* (*MYOD1*), *MYOG* and *PAX7*, were induced after 18-30 days of MYO induction, whereas expression of a DM marker, *ALX4*, was suppressed (Fig. 3E). The induction efficiency was estimated as approximately 22% based on the number of MYOD- and MYOG-positive cells (Fig. 3F).

The other derivative of DM is D, which gives rise to the dermis of the back, for which induction protocols have not been established. We first found that an extended culture period without passage for more than 11 days under the DM induction condition decreased the expression levels of *PAX3* and paraxis (DM and SM markers) and *PAX7* and *NCAD* (*CDH2*) (DM and MYO markers) within 6 days (Fig. 3G). The D can be defined as PDGFRα^+^/EN1^+^ cells, as PDGFRα is expressed in the D and dermal fibroblasts (DFs) (Orr-Urtreger et al., 1992), and EN1 is expressed in the D and DM (Ahmed et al., 2006). Therefore, we examined the expression of *PDGFRA* and *EN1* by RT-qPCR and immunocytochemistry and found that these markers were induced at day 9 of D induction (Fig. 3G,H and Fig. S7). Expression levels of *ALX4* and *MSX1* (DM and D markers) and *EGFL6* and *COL1A2* (D and DF markers) were upregulated at day 9 of D induction, too (Fig. 3G and Fig. S7). FACS analysis with anti-PDGFRα and anti-EN1 antibodies revealed that 69.5±1.4% of D cells were PDGFRα^+^ and 92.7±0.4% of PDGFRα^+^ cells were EN1^+^ (Fig. 3I), demonstrating successful induction of D under this condition. We also examined the function of iPSC-derived D. One of the main functions of dermal fibroblast in the body is to secrete extracellular matrix (ECM) proteins, such as collagen and hyaluronic acid, which serve to preserve moisture in the skin and help to sustain the skin structure. Therefore, we examined the amount of collagen type I and hyaluronic acid protein in the culture medium of iPSC-derived D and adult DF, and found that nearly the same amount of these ECM proteins was present in the both media (Fig. 3J).

These results indicate that continuous culture under a DM-inducing conditions for 12 days from SM directed the cells towards the D fate, whereas only the CHIR condition significantly reduced the percentage of PDGFRα^+^/EN1^+^ cells (data not shown). This biased fate suggests a reduction in MYO differentiation capacity even after 3 days of culture (day 11 in Fig. 3A). To assess this possibility, we compared the effect of different DM-inducing conditions on MYO differentiation. First, we cultured SM with CHIR99021 alone, BMP4 alone, CHIR99021 with low BMP4 (1 ng/ml), or CHIR99021 with high BMP4 (CB) for 3 days, and then cultured the cells with CHIR99021 for 30 days to induce myogenic differentiation. The expression of *PAX3*, which is expressed in primitive DM but not in D, was analyzed on day 11 by RT-qPCR and expression of MYOD was analyzed on day 41 by immunocytochemistry. Although CHIR99021 alone efficiently induced *PAX3* and MYOD, we found that CHIR99021 with BMP4, regardless of the concentration, had greater induction effects (Fig. S8). Therefore, cells cultured under the CB condition contain primitive DM cells, which have myogenic potential, rather than committed D progenitors.

### Induction of chondrocytes, osteocytes and SYN through SCL

**Fig. 4.**
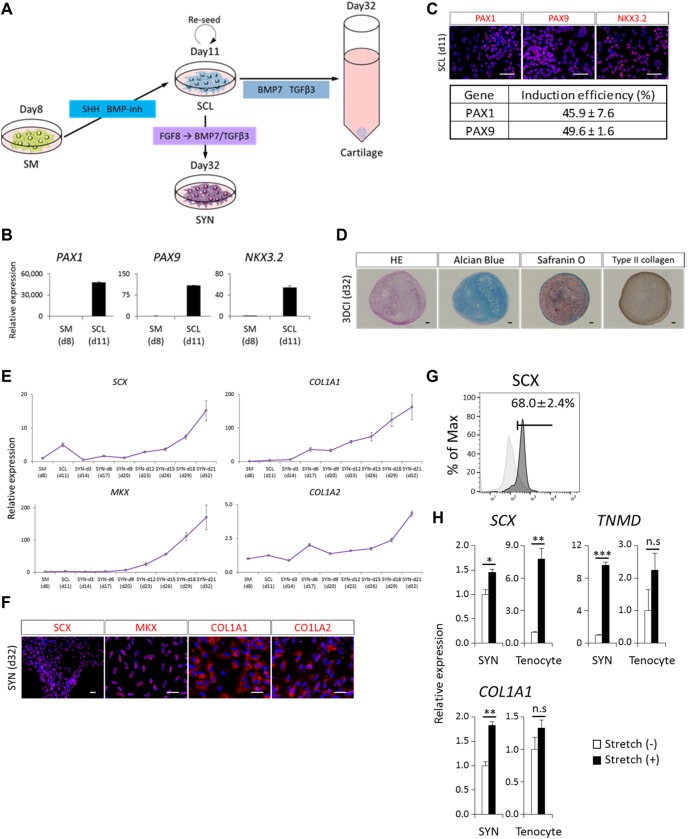
**Directed SM differentiation toward SCL and SYN fate.** (A) Schematic view of protocols for the induction of SCL and its derivatives (SYN and cartilage). For SCL induction, induced SM was treated with Smoothened agonist (SAG, 100 nM) and BMP inhibitor (LDN193189, 0.6 μM) for 3 days. For SYN induction, induced SCL was re-seeded onto a Matrigel-coated culture dish. Cells were treated with FGF8 (20 ng/ml) for 3 days and then a medium containing TGFβ3 (10 ng/ml) and BMP7 (10 ng/ml) until day 21 of the SYN induction. For 3DCI, induced SCL was detached from the dishes and transferred into a 15-ml conical tube with CI basal media supplemented with TGFβ3 (10 ng/ml) and BMP7 (10 ng/ml) for 21 days. (B,C) Differentiation toward SCL fate was assessed by RT-qPCR (B) and immunocytochemistry (C) at the indicated days (d). (D) Immunohistochemistry analysis of type II collagen expression and Hematoxylin and Eosin (HE), Alcian Blue and Safranin O staining in a 3DCI pellet at day 21 of 3DCI. (E-G) Differentiation toward SYN fate was assessed by RT-qPCR (E), immunocytochemistry (F) and FACS (G). The mean±s.e.m. from three sets of experiments are shown. iPSCs were used as control populations (G). (H) Effect of mechanical stretch stimulation on induced SYN was assessed by RT-qPCR. *P<0.05; **P<0.01; ***P<0.001 by Dunnett's multiple comparisons *t*-test compared with Stretch (-) (H). Error bars represent s.e.m. (*n*=3). Scale bars: 50 μm (C,F); 100 μm (D). 3DCI, three-dimensional chondrogenic induction; n.s, no significant difference.

The ventral side of the SM differentiates into SCL, an embryonic primordium of chondrocytes and osteocytes. It has been shown that SCL can be induced from SMs cultured with CDM supplemented with 100 nM SAG, a SHH activator, and 0.6 µM LDN193189, a BMP inhibitor (Zhao et al., 2014) (Fig. 4A). Consistent with previous reports, *PAX1*, *PAX9* and *NKX3*-*2* were induced directly from the induced SM within 3 days (Fig. 4B,C). The estimated induction ratio was approximately 45% based on immunocytochemistry with anti-PAX1 and anti-PAX9 antibodies. To assess the differentiation properties of SCL in the cartilage, induced SCL cells were collected, centrifuged, and cultured with three-dimensional (3D) chondrogenic differentiation medium (Hino et al., 2015). After 21 days of induction, the cells differentiated into chondrocytes, as demonstrated by Alcian Blue staining, Safranin O staining, immunohistochemistry for anti-type II collagen antibody (Fig. 4D), and RT-qPCR for chondrogenic markers (Fig. S9A). Additionally, the differentiation potential of SCL to osteocytes was revealed by RT-qPCR and Alizarin Red staining (Fig. S9B,C).

The medial part of the SCL has been defined as the SYN, which is a primordium of tendons and ligaments (Brent et al., 2003) and for which induction protocols from human PSCs have not been established. Because it was shown that FGF8 signaling is required for SYN development in the early phase (Brent et al., 2003) and that BMP and TGFβ signaling are involved in the development, maintenance and healing of tendons and ligaments (Pryce et al., 2009; Lee et al., 2011; Schwarting et al., 2015), we administered FGF8 for 72 h and then BMP7 and TGFβ3 without passage. In the later phase of induction, the expression of the SYN markers *SCX*, *MKX*, *COL1A1* and *COL1A2* was upregulated in a time-dependent manner (Fig. 4E), and the intensity was comparable to that of primary tenocytes (Fig. S10). Additionally, protein expressions were confirmed by immunocytochemistry at day 21 of SYN induction (Fig. 4F), and induction efficiency was estimated to be 68.0±2.4% by FACS (Fig. 4G). Regarding the function of induced SYN, some studies have reported that mechanical stress affects tendon development before and after birth and promotes the differentiation of tenocytes from precursor cells (Suzuki et al., 2016; Marturano et al., 2013). Thus, we evaluated the effects of mechanical stress on the culturing SYN. The results showed that iPSC-derived SYN and human primary tenocytes upregulated SYN-related markers in response to mechanical stress, consistent with previous reports (Fig. 4H) (Suzuki et al., 2016; Yang et al., 2004). These data demonstrate one aspect of the function of iPSC-derived SYNs.

Taken together, induced SMs can be differentiated into the SM derivatives D, MYO, SCL and SYN. To evaluate the induction protocols established in this study, gene expression profiles of the derivatives were assessed. A principal component analysis plot indicated the stepwise differentiation of each derivative, supporting the rationale of each procedure (Fig. S11).

### Induction of MSC-like cells from SMs

**Fig. 5.**
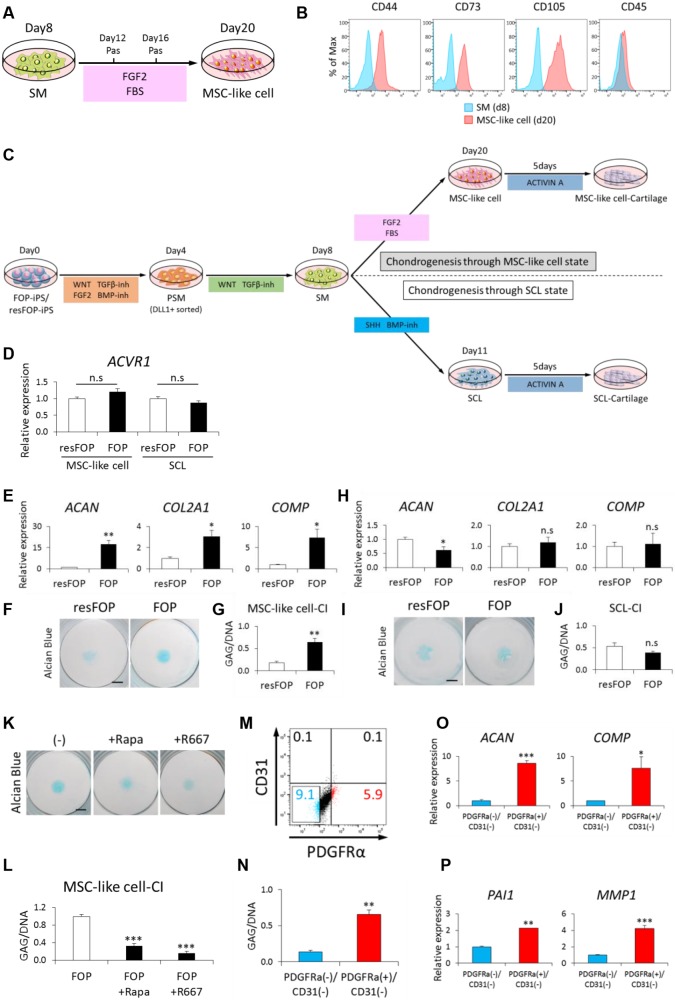
**Recapitulation of FOP phenotype shown by two chondrogenesis pathways.** (A) Schematic view of a protocol for MSC-like cell induction. Induced SM were treated with FGF2 (4 ng/ml) and FBS (10%) for 12 days. (B) FACS analysis using surface markers for MSCs at day 12 of MSC-like cell induction. CD44^+^, CD73^+^, CD105^+^ and CD45 (PTPRC)^−^ cells were induced. SM cells were used as control populations. (C) Schematic view of MSC-like cell-CI and SCL-CI using FOP-iPSCs and resFOP-iPSCs. Briefly, induced MSC-like cells and SCL were spotted onto fibronectin-coated dishes and treated with CI basal media supplemented with activin A (30 ng/ml) for 5 days. (D) *ACVR1* expression in MSC-like cells and SCL induced from both FOP-iPSCs and resFOP-iPSCs was assessed by RT-qPCR. Error bars represent s.e.m. (*n*=6). The expression level of resFOP-MSCs was set to 1. (E-G) Evaluation of MSC-like cell-CI using FOP-iPSCs and resFOP-iPSCs. Chondrogenic differentiation was assessed at day 5 of CI by RT-qPCR analysis (E), Alcian Blue staining (F) and GAG/DNA analysis (G). (H-J) Evaluation of SCL-CI using FOP-iPSCs and resFOP-iPSCs. Chondrogenic differentiation was assessed at day 5 of CI by RT-qPCR analysis (H), Alcian Blue staining (I) and GAG/DNA analysis (J). (K,L) Evaluation of R667 and Rapamycin efficacy on MSC-like cell-CI. Chondrogenic differentiation was assessed at day 5 of CI by Alcian Blue staining (K) and GAG/DNA analysis (L). (M-P) *In vitro* study regarding cell-of-origins of the ectopic bone observed in FOP. PDGFRα^+^/CD31^–^ and PDGFRα^–^/CD31^–^ populations were isolated by FACS (M). Chondrogenic potential of each population was assessed at day 5 of CI by GAG/DNA (N) and RT-qPCR analysis (O). Expression levels of *PAI1* and *MMP1*, both surrogate markers of aberrant FOP-ACVR1 signaling, were higher in PDGFRα^+^/CD31^–^ cells (P). Error bars represent s.e.m. (*n*=3). *P<0.05; **P<0.01; ***P<0.001 by Student's *t*-test compared with resFOP (D,E,G,H,J), FOP (L) and PDGFRα^–^/CD31^–^population (N,O,P). n.s, no significant difference. Scale bars: 200 μm. Pas, passage; R667, R667 10 nM; Rapa, rapamycin 10 nM.

MSCs are of a postnatal cellular origin of bone, cartilage and adipose tissue and characterized as positive for MSC surface markers (Dominici et al., 2006; Awaya et al., 2012; Mahmood et al., 2010). To address whether SMs can differentiate into cells compatible with MSCs, SMs were cultured with 10% fetal bovine serum (FBS)/α minimum essential medium (αMEM) supplemented with FGF2 (Fig. 5A), as our previous report showed that this medium can induce MSCs from neural crest cells (NCCs) (Fukuta et al., 2014). After two passages, the cells changed their morphology to fibroblast-like and became positive for the MSC markers CD44, CD73 (NT5E) and CD105 (ENG) (Fig. 5B and Fig. S12A). The differentiation capacity of the somite-derived MSC (SMMSC)-like cells into osteocytes, chondrocytes and adipocytes was confirmed using standard procedures (Fig. S12B). SCL, a derivative of SM, can also undergo osteogenesis and chondrogenesis. To discriminate SCL and MSC-like cells, hierarchical cluster analysis was performed, and the result suggested that iPSC-derived SMMSC-like cells and human primary adult bone marrow MSCs were grouped together, whereas iPSC-derived SCLs were not; thus, iPSC-derived SMMSC-like cells are more similar to adult state bone marrow MSCs than are iPSC-derived SCLs (Fig. S13). Additionally, the expression of each marker was assessed by RT-qPCR analysis and microarray. Consequently, induced MSC-like cells were positive for the MSC markers *CD44*, *CD73* and *CD105*, but not for markers of SM or SCL (Figs S14 and S15), and the expression intensity was nearly the same as that of primary bone marrow MSCs (Fig. S15). These results indicate that the induced SMMSC-like cells were compatible with primary adult MSCs.

### Enhanced chondrogenesis in FOP-iPSC-derived MSC-like cells, but not in FOP-iPSC-derived SCL

One of the promising uses of iPSCs is for disease modeling with patient-specific iPSCs. Because we successfully induced two different types of chondrocytes, SCL-derived and MSC-like cell-derived, we applied the protocols to an osteochondral disorder caused by genetic mutations. FOP is an intractable rare disease characterized by endochondral ossification in patients' soft tissues mainly after birth. Therefore, we predicted that MSC-like cell-derived chondrocytes from FOP-iPSCs would enhance chondrogenesis as shown in our previous published study using neural crest-derived MSCs (Matsumoto et al., 2015; Hino et al., 2015), whereas SCL-derived embryonic chondrocytes would not have this effect. SMs were induced from FOP-iPSCs and gene-corrected (rescued) FOP-iPSCs (resFOP-iPSCs), which are derived from two FOP patients (patients 1 and 2 described in the Materials and Methods) (Matsumoto et al., 2015; Hino et al., 2015). MSC-like cells and SCL were induced according to the protocol described above (Figs S16 and S17) and subjected to two-dimensional (2D) chondrogenic differentiation with chondrogenic medium supplemented with or without activin A, a stimulator of mutant ACVR1 (Hino et al., 2015) (Fig. 5C). Although the expression levels of *ACVR1* were similar regardless of the cell type and mutation (Fig. 5D), only FOP-MSC-like cells stimulated with activin A showed enhanced chondrogenesis in terms of chondrogenic marker expression and glycosaminoglycan (GAG) production (Fig. 5E-J and Fig. S18, S19). We confirmed similar expression levels of SCL markers such as *PAX1* and *PAX9* in resFOP-SCL and FOP-SCL (Figs S16B and S17B). R667, a retinoic acid receptor-γ agonist, and rapamycin, an mTOR inhibitor, both of which are confirmed potent inhibitors of heterotopic ossification (Hino et al., 2017), attenuated the enhanced chondrogenesis (Fig. 5K,L and Fig. S20A). Recently, one cell-of-origin of the FOP lesion was proposed as PDGFRα^+^/CD31^−^ cells (Dey et al., 2016). Therefore, PDGFRα^+^/CD31^−^ cells and PDGFRα^−^/CD31^−^ cells were sorted from FOP-MSC-like cells by FACS (Fig. 5M and Fig. S21) and subjected to 2D chondrogenic differentiation. The expression levels of *ACVR1* and *PDGFRA* in isolated cells were assayed by RT-qPCR (Fig. S20B,C). As expected, PDGFRα^+^/CD31^−^ cells showed enhanced chondrogenesis compared with PDGFRα^−^/CD31^−^ cells (Fig. 5N,O). Interestingly, the expression levels of *PAI1* (*SERPINE1*) and *MMP1*, indicator genes of mutant ACVR1 activation (Matsumoto et al., 2015), were upregulated in PDGFRα^+^/CD31^−^ cells (Fig. 5P). These results indicate the cell-type specificity of FOP phenotypes and that our protocols can be used for disease modeling, phenotype analyses, and drug discovery.

## DISCUSSION

**Fig. 6.**
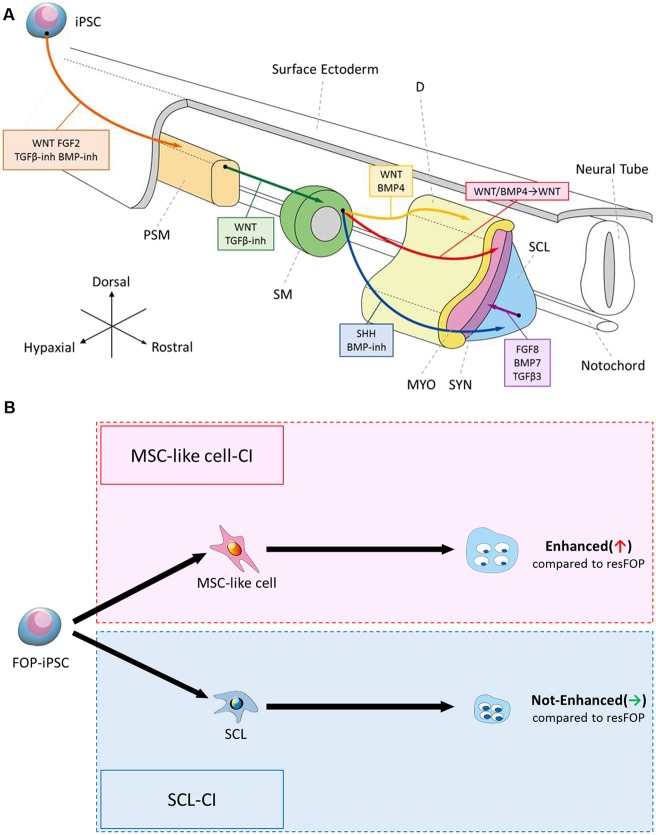
**Schematic summary of this study.** (A) Recapitulation of human somitogenesis and somite patterning *in vitro* using human iPSCs. We generated four SM derivatives from human iPSCs in a stepwise manner by referring to these signaling environments during mouse and chick somite development as shown on the diagram. (B) Recapitulation of the FOP phenotype in two chondrogenesis pathways. Chondrogenesis was enhanced in the MSC-like cell-CI pathway but not in the SCL-CI pathway.

Several studies have reported the induction of SMs and their derivatives from human and mouse PSCs. Typically, reports have focused on specific SM derivatives, such as skeletal muscle, bone and cartilage (Loh et al., 2016; Chal et al., 2018, 2015; Xi et al., 2017; Zhao et al., 2014; Umeda et al., 2012; Sakurai et al., 2009), and some of these differentiation processes have been carried out by forced expression of key genes, resulting in a new understanding of the genetic regulation of cell types (Tanaka et al., 2013). In the present study, we established induction protocols from PSCs to four SM derivatives, D, MYO, SCL and SYN cells, under the specific conditions determined for each by referring to studies of mouse and chick development (Fig. 6A). Specific signals favored the generation of specific cell types. This is the first report to demonstrate the successful induction of D and SYN from PSCs using SMs. We also showed that SMs can be differentiated into MSC-like cells *in vitro* for the first time, although experiments using lineage-traced mice are necessary to evaluate whether SM is an *in vivo* source of MSCs.

Three major studies have developed induction protocols for SMs from human iPSC-derived PSMs (Loh et al., 2016; Chal et al., 2015; Xi et al., 2017). In previously published protocols and the present report, the cells were initially cultured with activin/nodal/TGFβ signaling inhibitors, which induces the neural (dorsal) fate, and with WNT signaling activators. The successful derivation of SM suggests that the paraxial mesoderm belongs to the dorsal region and that WNT signaling plays a pivotal role in changing the fate from neural to paraxial mesoderm. We also monitored key alternative cell fates (LPM and NCC) in the iPSC-derived PSM and SM states (Figs S22, S23, S24), as these lineages are known to generate MSC-like cells under certain conditions. iPSC-derived LPM and NCC were acquired using previously published protocols (Iyer et al., 2015; Fukuta et al., 2014). The results showed that our induction methods preferentially lead iPSCs toward the paraxial mesoderm lineage, but not the LPM or NCC lineages. Thus, the SM derivatives induced in this work, e.g. D, SYN, SMMSC-like cell, and others, were mainly derived from the paraxial mesoderm, but not from the LPM or NCC. Furthermore, in this study, we used CHIR99021 during SM differentiation as a WNT pathway activator because several WNTs are expressed in the surrounding tissues of the SM and the Wnt-reporter is active in the SM (Maretto et al., 2003). The results showed that epithelialization was observed only in S10C5 based on the accumulation of CDH11 in cell-cell junctions (Fig. 2G). This observation indicates the involvement of WNT signaling in SM epithelialization, and our protocol may better recapitulate the endogenous signaling environment. Moreover, we evaluated the differentiation properties of SM induced under several WNT activities and found that only SM induced with CHIR99021 highly expressed the DM marker *ALX4* and MYO markers *MYOD* and *MYOG* after DM and MYO induction, respectively (Fig. S25). Interestingly, higher levels of CHIR99021 (10 µM) repressed PAX3-GFP expression (Fig. 2B,C and Fig. S5), but no contamination of LPM or NCC was observed regardless of the CHIR99021 concentration (Fig. S24). Taken together, these results suggest that functional SMs were differentiated following CHIR99021 administration and that the proper level of WNT activity during SM induction is crucial.

Our stepwise protocols using CDM can be used to determine the different differentiation requirements of the signaling environment and provide important insights into human SM development. For example, retinoic acid, a metabolite of vitamin A, plays a pivotal role in hindbrain and SM development, and its disruption induces developmental toxicity in human embryogenesis (Kam et al., 2012; Rhinn and Dolle, 2012). Our SMs could be used to analyze the toxicity of retinoic acid on human somitogenesis. Another characteristic of the paraxial mesoderm is mesenchymal-to-epithelial transition and subsequent epithelial-to-mesenchymal transition. Notably, in mouse, chick and zebrafish, mesenchymal-to-epithelial transition is regulated by oscillatory gene expression in the PSM (Hubaud and Pourquié, 2014; Jiang et al., 2000; Jouve et al., 2002), but a lack of experimental tools has prevented confirmation in humans. Our stepwise induction protocols should be useful for such research.

There were discrepancies between our results and those of previous *in vivo* studies. Experiments using mouse and chick showed that BMP induces the lateral DM and that WNT induces the medial DM (Marcelle et al., 1997; Ikeya and Takada, 1998; Hirsinger et al., 1997). Under our DM induction conditions, however, both BMP4 and CHIR99021 (CB condition) successfully induced *ALX4*, *EN1* and noggin (Fig. 3B). One possible explanation for the difference is that in our defined culture condition it is difficult to induce specific parts of the DM because some components are unknown. The culture conditions that induce the dorsal and ventral DM require further analysis.

We applied our protocols to disease modeling of FOP, a rare intractable disease characterized by heterotopic endochondral ossification in patients' soft tissues mainly in childhood. Notably, FOP-iPSC-derived MSC-like cells showed enhanced chondrogenesis, whereas FOP-iPSC-derived SCL, a major embryonic origin of the axial skeleton, did not have this effect, despite the addition of activin A, a stimulator of mutant ACVR1. These data indicate the cell-type specificity of the FOP phenotypes and possibly reflect the postnatal onset of the disease (Fig. 6B). To demonstrate fully the advantage of our stepwise differentiation protocols, we also compared the chondrogenic capacity of MYO, D and SYN induced from FOP and resFOP iPSCs. These cells rarely expressed chondrogenic markers such as *ACAN*, *COL2A1* and *COMP* compared with MSC-like cell-derived chondrocytes (data not shown), suggesting their low chondrogenic potential. By using FOP mouse models, one study showed that mouse myofiber and myoprogenitor lineage cells marked by *Myf6-Cre* did not contribute to ectopic bone formation, which is consistent with our observations (Dey et al., 2016). However, the same study detected the contribution of tendon progeny cells marked by *Scx-Cre*, which is inconsistent with our observations. Because of technical limitations *in vitro*, we cannot rule out the possibility that enhanced chondrogenesis in MYO and SYN was not observed because of the inadequate culture periods and/or conditions, e.g. tendon progeny should be differentiated into MSCs before starting chondrogenesis. Additionally, in this study we used iPSCs harboring a classic FOP mutation (c.617G>A; p.R206H). However, there are several non-classic type mutations that result in phenotypic variations in terms of the severity and onset of disease. Further analyses are needed to generalize our findings to the FOP disease state.

Another important application of the induced SM derivatives is for regenerative medicine. The derivatives may be applicable for musculoskeletal disorders, such as muscular dystrophy, articular cartilage defects, bone defects and tendon ruptures. One of the advantages of our protocols is the use of chemically defined medium for all induction procedures except for MSC-like cell induction. Although in this study we used iPSCs maintained with feeder cells and Matrigel as a surface coat on the dish during induction, preliminary experiments using feeder-free/xeno-free iPSCs and iMatrix-coated dishes also showed high induction efficiencies from iPSCs to SMs through PSMs (Figs S1B,C, S26 and S27). A screening experiment to examine PSM-inducing conditions using several iPSC lines indicated the cell-type specificity of CHIR99021 sensitivity and the necessity of determining the culture conditions for each cell lines (Fig. S26). Xeno-free conditions can likely be used for other induction protocols as well. Cell quantity and quality, which include the purity and maturation of the desired cells, must also be improved. For example, in heart regeneration, 1×10^9^ cardiomyocytes are predicted to be needed for engraftments that recover function in large non-human primates (Chong et al., 2014). For tendon/ligament regeneration, not only the cell number but also the cell strength are important. Improvements in induction efficiency, the development of surface markers to purify each cell type, and methods for 3D reconstitution are needed to advance our protocols for clinical cell-based therapies.

## MATERIALS AND METHODS

### Cell culture

Human iPSCs were maintained on SNL feeder cells in primate ES cell medium (ReproCELL) supplemented with 4 ng/ml FGF2 (Wako) as described previously (Takahashi et al., 2007). All figures show data from 201B7-PAX3-GFP iPSCs, in which EGFP replaces one allele of the *PAX3* coding sequence in exon 1. Generation of 201B7-PAX3-GFP iPSCs will be described elsewhere (H. Sakurai, personal communication), except for Fig. 5, Figs S16-S21 (FOP-iPSCs and resFOP-iPSCs), and Figs S1, S2, S26 and S27 (several wild-type iPSC lines).

Because *Pax3^GFP/+^* heterozygous mice with the same knock-in design were viable and fertile and GFP expression recapitulates endogenous *Pax3* expression in mice (Lagha et al., 2010), we employed the same knock-in design. In Fig. S2, various iPSC lines (201B7, TIG118-4f, 414C2, 409B2 and 1231A3) were used to examine the reproducibility of this study (Koyanagi-Aoi et al., 2013; Okita et al., 2011; Takahashi et al., 2007; Nakagawa et al., 2014). As shown in Fig. 5, FOP-iPSCs harboring the c.617G>A (p.R206H) heterozygous mutation in *ACVR1* and gene-corrected resFOP-iPSCs generated by BAC-based homologous recombination were used. FOP-iPSCs and resFOP-iPSCs were prepared from two patients, patients 1 and 2, previously described as ‘patient 1 FOP clone 1’ and ‘patient 1 resFOP clone 1’, and ‘patient 2 FOP clone 1’ and ‘patient 2 resFOP clone 1’ (Hino et al., 2015). All experiments shown in Fig. 5 were performed using FOP-iPSCs and resFOP-iPSCs from patient 1.

### PSM induction from hiPSCs

Prior to PSM induction from hiPSCs, SNL feeder cells were removed and iPSCs were seeded onto Matrigel (BD Biosciences)-coated dishes (1.3×10^6^ cells/10-cm dish). After 3 days of feeder-free culture in mTeSR1 medium (STEMCELL Technology), the cells were cultured with PSM induction medium [CDM basal medium (Iscove's modified Dulbecco's medium/Ham's F-12 1:1 (Gibco), 1× chemically defined lipid concentrate (Gibco), 15 mg/ml apo-transferrin (Sigma), 450 mM monothioglycerol (Sigma), 5 mg/ml purified bovine serum albumin (99% purified by crystallization; Sigma), 7 mg/ml insulin (Wako) and penicillin/streptomycin (Invitrogen)] supplemented with 10 μM SB431542 (Sigma), 10 μM CHIR99021 (Wako), 2 μM DMH1 (Tocris) and 20 ng/ml FGF2 for 4 days. The induction medium was changed on day 3. Induction efficiency was determined by FACS with anti-DLL1 antibody and PAX3-GFP.

### SM induction from PSM

A total of 1.0×10^5^ DLL1^+^ cells were seeded into one well of a Matrigel-coated 12-well plate and subjected to SM induction. SM induction was carried out for 4 days in CDM basal medium supplemented with 10 μM SB431542 and 5 μM CHIR99021. The induction medium was changed on day 3 of SM induction.

### DM induction from SM

DM induction was carried out by replacing the SM induction medium with DM induction medium [5 μM CHIR99021 and 10 ng/ml BMP4 (R&D Systems) in CDM basal medium]. DM induction was performed for 3 days, and the induction medium was changed on day 2. The composition of the DM induction medium was determined by mini-screening of 25 conditions using various concentrations of CHIR99021, IWR1 (Cayman Chemical), BMP4 (R&D Systems) and DMH1 (Tocris).

### MYO induction from DM

MYO induction was carried out by replacing the DM induction medium with MYO induction medium (5 μM CHIR99021 in CDM basal medium). MYO induction was performed for 30 days, and the induction medium was changed every 3 days.

### D induction from DM

D induction was carried out by culturing of cells in DM induction medium. D induction was performed for 9 days, and the induction medium was changed every 3 days.

### SCL induction from SM

SCL induction was carried out by replacing the SM induction medium with SCL induction medium [100 nM SAG (Calbiochem) and 0.6 μM LDN193189 (Stemgent) in CDM basal medium], as described previously (Zhao et al., 2014). SCL induction was performed for 3 days, and the induction medium was changed on day 2.

### SYN induction from SCL

Induced SCL was detached from the dish with 0.25% trypsin-EDTA (Gibco), and a total of 5.0×10^4^ cells were seeded into one well of a Matrigel-coated 24-well plate and subsequently subjected to SYN induction. The first step of SYN induction was carried out for 3 days in CDM basal medium supplemented with 20 ng/ml FGF8 (Peprotech). The medium was replaced with second step SYN induction medium [10 ng/ml BMP7 (R&D Systems) and 10 ng/ml TGFβ3 (R&D Systems) in CDM basal medium]. The second step of SYN induction was performed for 18 days, and the induction medium was changed every 3 days.

### MSC-like cell induction from SM

MSC-like cell induction was carried out by replacing SM induction medium with MSC-like cell induction medium [10% FBS (Nichirei) and 4 ng/ml FGF2 (Wako) in αMEM (Nacalai Tesque)]. Passage was performed every 4 days using 0.25% trypsin-EDTA (Gibco). Cells were seeded into a tissue culture dish at a density of 2×10^4^ cells/cm^2^. MSC-like cell induction was performed for 12 days, and marker expressions were assessed by FACS on day 12 of MSC-like cell induction. Osteogenic induction (OI), chondrogenic induction (CI) and adipogenic induction (AI) were performed to confirm the differentiation potencies of the induced MSC-like cells. CI was performed as described below. OI, AI and Alizarin Red, Alcian Blue and Oil Red O staining were performed as described previously (Fukuta et al., 2014).

### 2D chondrogenic induction (2DCI)

A total of 1.5×10^5^ induced MSC-like cells or SCL were suspended in 5 μl of chondrogenic basal medium [DMEM: F12 (Invitrogen), 1% (v/v) ITS+Premix (BD Biosciences), 0.1 μM dexamethasone (Wako), 0.17 mM L-ascorbic acid 2-phosphate sesquimagnesium salt hydrate (Sigma), 0.35 mM proline (Sigma), 0.15% (v/v) glucose (Sigma), 1 mM sodium pyruvate, 2 mM GlutaMax (Invitrogen) and 1% (v/v) FBS], and subsequently transferred to one well of fibronectin-coated 24-well plates (BD Biosciences). After 1 h of incubation at 37° C in 5% CO_2_, the cells formed a micromass. Next, we added 1 ml of chondrogenic basal medium supplemented with 10 ng/ml BMP7 (R&D Systems) and 10 ng/ml TGFβ3 (R&D Systems). For phenotype analyses of FOP, we added chondrogenic basal medium supplemented with 30 ng/ml activin A (R&D Systems) with/without 10 nM R667 (Toronto Research Chemicals), or with 30 ng/ml activin A with/without 10 nM rapamycin (MedChem Express). The micromass was cultured at 37°C in 5% CO_2_ for 5 days. Differentiation properties were assessed by RT-qPCR analysis, glycosaminoglycans (GAG) quantification, and Alcian Blue staining as described previously (Hino et al., 2015; Umeda et al., 2012; Craft et al., 2015; Oldershaw et al., 2010). Briefly, induced cells were fixed for 30 min with 4% paraformaldehyde (Wako), rinsed with PBS, and then stained overnight with Alcian Blue solution (1% Alcian Blue, pH 1) (Muto Pure Chemicals).

### 3D chondrogenic induction (3DCI)

A total of 1.0×10^6^ induced SCL cells were suspended in 0.5 ml of chondrogenic basal medium supplemented with 10 ng/ml BMP7 (R&D Systems) and 10 ng/ml TGFβ3 (R&D Systems), transferred into 15-ml tubes (Corning), centrifuged for 5 min at 280 ***g*** to form pellets, and incubated at 37°C in 5% CO_2_ for 21 days. The culture medium was changed every 3 days.

### 2D osteogenic induction (2DOI) from SCL

A total of 4.0×10^5^ induced SCL cells were seeded into a Matrigel-coated 12-well plate and subsequently subjected to 2DOI by using MSCgo Rapid Osteogenic Medium (Biological Industries).

### Human primary samples

Human dermal fibroblasts were purchased from Cell Application and cultured in DMEM supplemented with 10% FBS. Human anterior cruciate ligament samples were purchased from Articular Engineering and total RNA was extracted after homogenization. Human tenocytes were purchased from Angio-Proteomie and cultured in Tendon Cell Growth Medium (Angio-Proteomie).

### RT-qPCR analysis

Total RNA was puriﬁed with an RNeasy Kit (Qiagen) and treated with a DNase-one Kit (Qiagen) to remove genomic DNA. Reverse transcription was carried out using 1 μg of total RNA and Superscript III reverse transcriptase (Invitrogen), according to the manufacturer's instructions. RT-qPCR was carried out with Thunderbird SYBR qPCR Mix (Toyobo) and analyzed using the QuantStudio12K Flex real-time PCR system (Applied Biosystems) or StepOne real-time PCR system (Applied Biosystems). Primer sequences are shown in Table S1.

### Immunocytochemistry, immunohistochemistry and histological analyses

Prior to performing immunocytochemistry with antibodies, the cells on plates were fixed with 2% paraformaldehyde at 4°C for 10 min, washed twice with PBS, incubated with 0.2% methanol (Nacalai Tesque) or 0.2% Tween 20 (Sigma)/PBS at 4°C for 15 min as the surface-active agent for penetration processing, and treated with Blocking One (Nacalai Tesque) or 1% bovine serum albumin/PBS at 4°C for 1 h. Cells were then treated with primary antibodies at 4°C overnight. Next, the samples were washed several times with 0.2% Tween 20/PBS and incubated with secondary antibodies for 1 h at room temperature. DAPI (1:5000; Sigma) was used to counterstain the nuclei. The primary and secondary antibodies used in this study are listed in Table S2. Immunohistochemistry of anti-type II collagen antibody and histological analyses, such as Hematoxylin and Eosin staining, Alcian Blue staining and Safranin O staining of induced 3DCI pellets, were carried out by the Center for Anatomical, Pathological and Forensic Medical Researches, Graduate School of Medicine, Kyoto University. Samples were observed and assessed using a BZ-X700 fluorescence microscope (Keyence, Osaka, Japan). With respect to the immunocytochemistry of myosin heavy chain (MHC), images were acquired using the optical sectioning system of the BZ-X700.

### FACS and analysis

FACS was performed using AriaII (BD Biosciences) according to the manufacturer's protocol. The antibodies used in FACS are listed in Table S3. Intracellular flow cytometry analysis was also performed with the AriaII (BD Biosciences) according to the manufacturer's protocol. Briefly, the cells were fixed and permeabilized prior to antibody staining. The antibodies used in intracellular flow cytometry analysis are listed in Table S2. The expression rate of each differentiation marker was calculated by comparing that of iPSCs or induced SM or DM, as described in each figure legend.

### GAG assay

GAG content in the pellets was quantified with the Blyscan Glycosaminoglycan Assay Kit (Biocolor). DNA content was quantified using the PicoGreen dsDNA Quantitation Kit (Invitrogen).

### Microarray analysis

Total RNA was prepared using the RNeasy Mini Kit (Qiagen). cDNA was synthesized using the GeneChip WT (Whole Transcript) Sense Target Labeling and Control Reagents Kit as described by the manufacturer (Affymetrix). Hybridization to the GeneChip Human Gene 1.0 ST expression arrays, washing, and scanning were performed according to the manufacturer's protocol (Affymetrix). Expression values were calculated using the RMA summarization method, and the data obtained were analyzed with GeneSpring GX 14.5 (Agilent Technologies) for PCA, hierarchical cluster analysis and gene expression analysis. PCA analysis was conducted using the expression values (two-fold higher with statistical significance). Statistical analyses were performed by one-way analysis of variance with a Benjamini and Hochberg False Discovery Rate (BH-FDR 50.01) multiple testing correction followed by Tukey honest significant difference post-hoc tests (GeneSpring GX).

### ELISA

iPSC-derived D and adult DF (1×10^5^) were seeded into 24-well plates and subsequently cultured in each culture medium. The culture medium was collected after 3 days, and the amounts of secreted protein were quantified by generating a standard curve by plotting the AlphaLISA counts aginst the concentration of the control analyte according to the manufacturer’s instructions (AlphaLISA COL1A1 detention kit and AlphaLISA Hyaluronic Acid detection kit, PerkinElmer).

### Mechanical stretch stimulation

iPSC-derived SYNs and tenocytes (1×10^5^) were seeded into multi-well type silicon rubber chambers (Strex) coated with Matrigel 24 h before stretching. The chambers were set on the device (Strex) and monoaxial cyclic strain (0.5 Hz, 5%) was forced for 12 h. After stretching, the cells were collected and total RNA was extracted for subsequent RT-qPCR analysis.

### Statistics

The statistical significance of all data was calculated using GraphPad Prism7. *P*-values less than 0.05 were considered as significant.

### Study approval

All experimental protocols involving patient samples were approved by the Ethics Committee of the Department of Medicine and Graduate School of Medicine, Kyoto University. Written informed consent was provided by each donor.

## Supplementary Material

Supplementary information
