# Supplementary material for: Modeling human somite development and fibrodysplasia ossificans progressiva with induced pluripotent stem cells
Source: Development. 2018 Aug 23;145(16):dev165431. doi: 10.1242/dev.165431 (PMC6124548; doi:10.1242/dev.165431)
Supplement: Supplementary information [file develop-145-165431-s1.pdf]

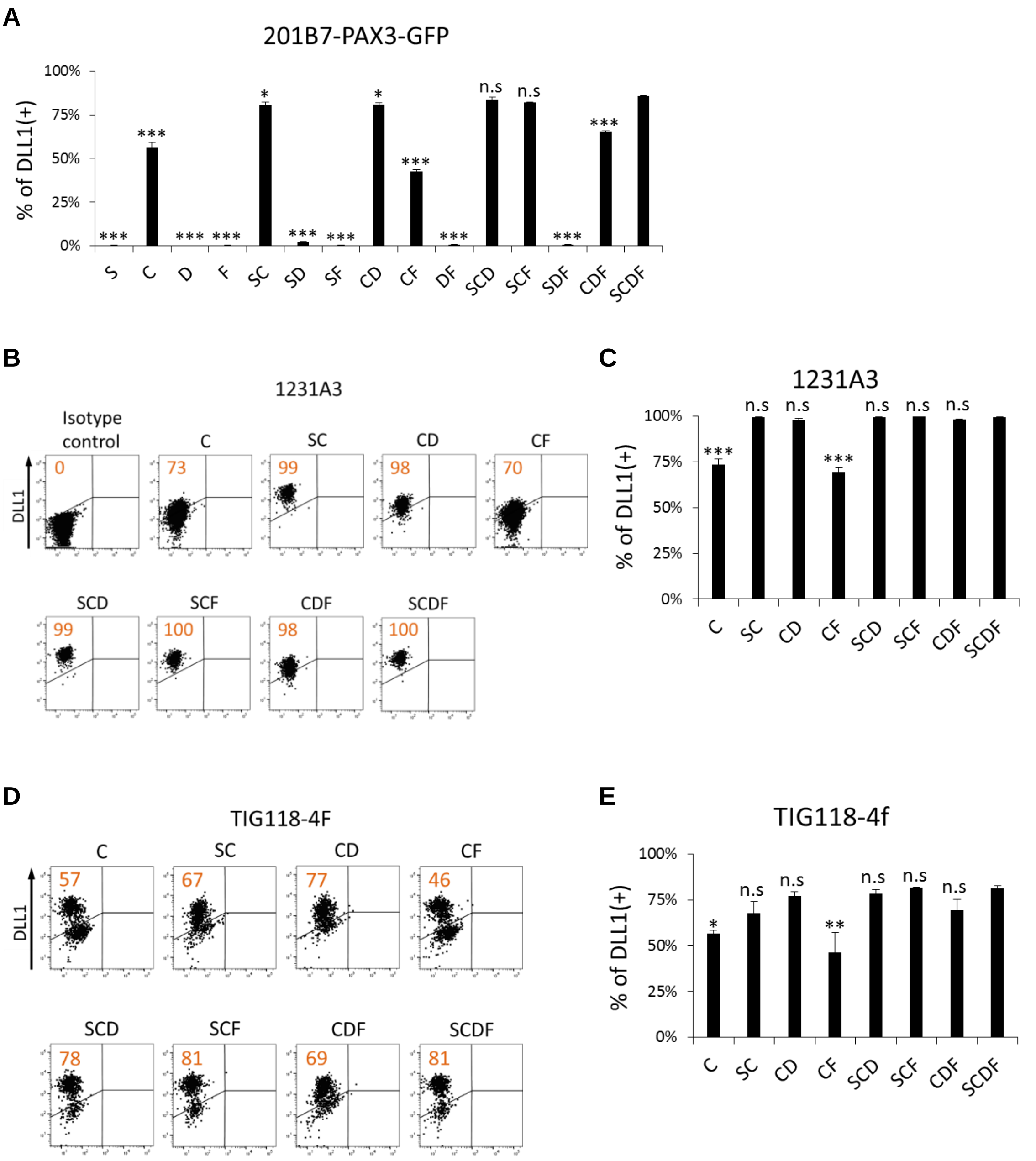

Figure S1. Investigation of optimal protocol for PSM induction.

(A-E) FACS analysis using anti-DLL1 antibody. 201B7-PAX-GFP **(A)**, 1231A3 **(B-C)** and TIG118-4f **(D-E)** iPSC lines were cultured in various conditions, and the induction efficiencies were assessed at day 4 by FACS. Error bars: mean ± SE (n=3). *p < 0.05; **p < 0.01; *** p< 0.001 by Dunnett's multiple comparisons *t*-test compared with SCDF. n.s, no significant difference.

Development • Supplementary information

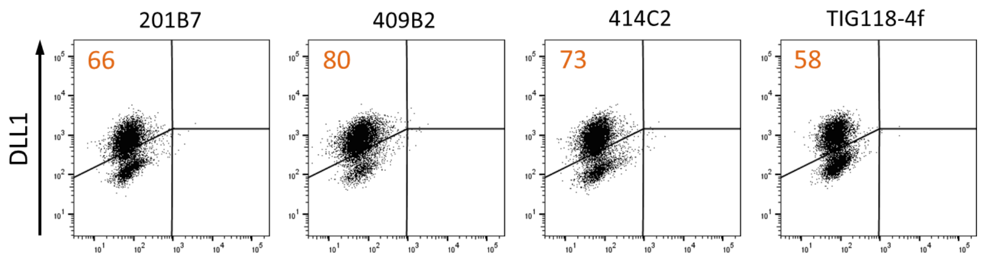

Figure S2. Induction of PSM with various iPSCs.

FACS analysis using anti-DLL1 antibody showed robustness of the induction protocol. 201B7, TIG118-4f, 414C2 and 409B2 iPSC lines were cultured in SCDF condition, and the induction efficiencies were assessed at day 4 by FACS.

Development • Supplementary information

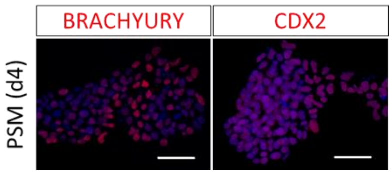

Figure S3. Immunocytochemistry analysis of induced PSM.

Immunocytochemistry analysis at day 4 of PSM induction. Cells were stained with anti-BRACHYURY and anti-CDX2 antibodies and co-stained with DAPI. Scale bars: 50 µm.

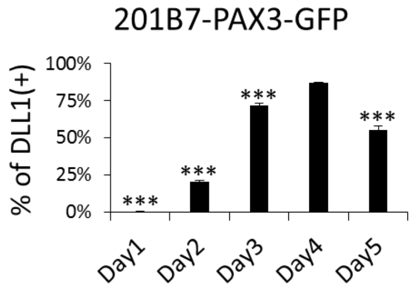

Figure S4. Investigation of optimal day for PSM induction.

FACS analysis using anti-DLL1 antibody. 201B7-PAX-GFP iPSCs were cultured in SCDF, and the induction efficiency was assessed from day 1 until day 5 by FACS. Error bars: mean ± SE (n=3). *** p< 0.001 by Dunnett's multiple comparisons *t*-test compared with day 4.

Development • Supplementary information

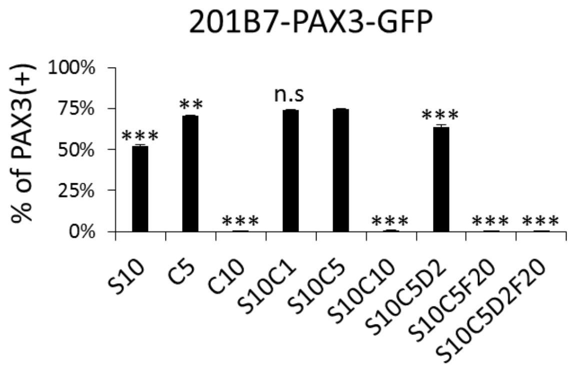

Figure S5. Investigation of optimal protocol for SM induction.

Characterization of SM induced by various conditions. The induction efficiencies were assessed by PAX3-GFP FACS analysis. Error bars: mean ± SE (n=3). **p < 0.01; *** p< 0.001 by Dunnett's multiple comparisons *t*-test compared with S10C5. n.s, no significant difference.

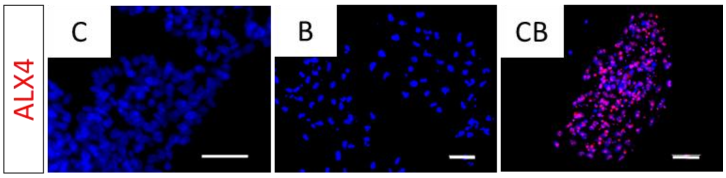

Figure S6. DM induction with CHIR99021 and BMP4.

Immunocytochemistry analysis at day 3 of DM induction. Cells were stained with anti-ALX4 antibody and co-stained with DAPI. Both CHIR99021 and BMP4 were necessary to induce DM. Scale bars: 50 µm. C, CHIR99021 5 µM; B, BMP4 10 ng/mL.

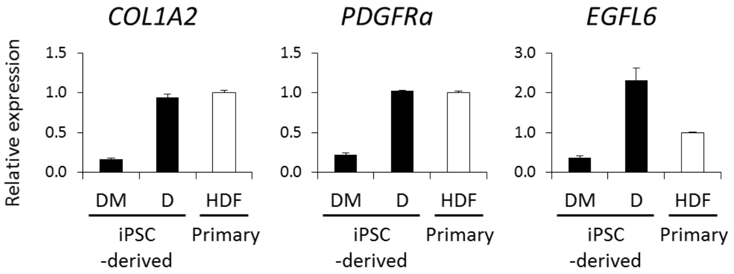

Figure S7. Comparative analysis of iPSC-derived dermatome and primary adult dermal fibroblast.

Gene expressions of dermatome and dermal fibroblast-related markers in induced D and primary HDFs were analyzed by RT-qPCR. Error bars: mean ± SE (n=3). HDF, human dermal fibroblasts.

Development • Supplementary information

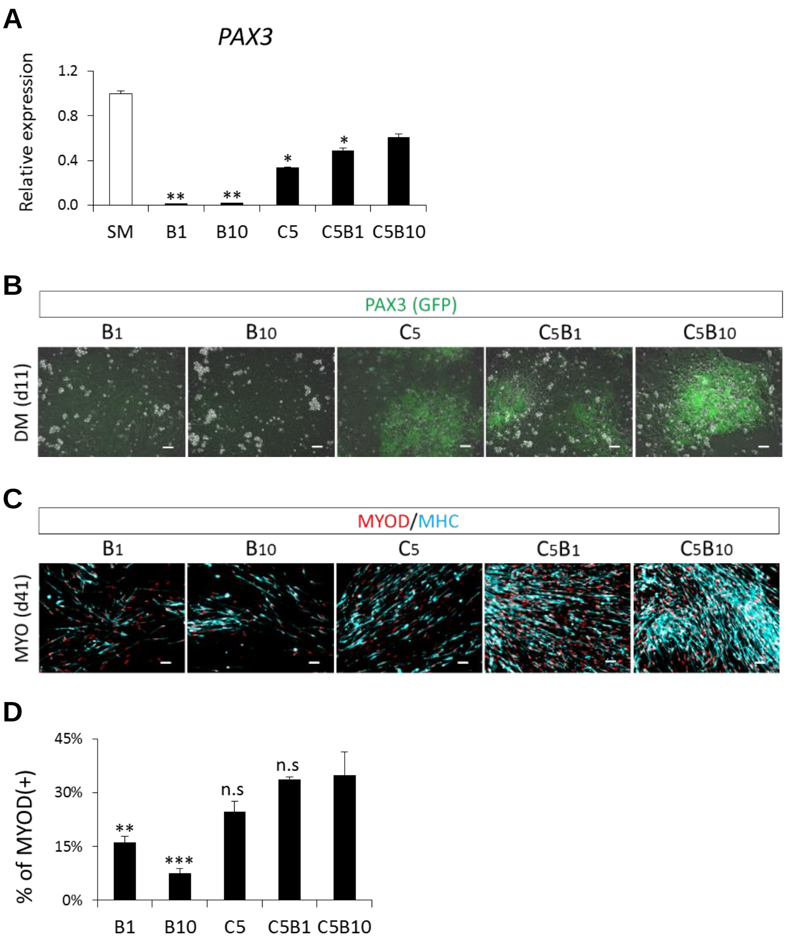

Figure S8. Optimal protocol for DM induction.

(A-B) Characterization of DM induced by various conditions. Quality of induced DM was assessed by RT-qPCR **(A)** and PAX3-GFP fluorescence **(B)** analyses.

(C-D) Characterization of MYO differentiated from DM. Quality of induced MYO was assessed by immunocytochemistry analysis **(C)**. Induction efficiencies **(D)**. Error bars: mean ± SE (n=3). *** $p < 0.001$; ** $p < 0.01$; * $p < 0.05$ by Dunnett's multiple comparisons *t*-test compared with C5B10 (A, D). n.s, no significant difference. Scale bars: 50 μm.

Development • Supplementary information

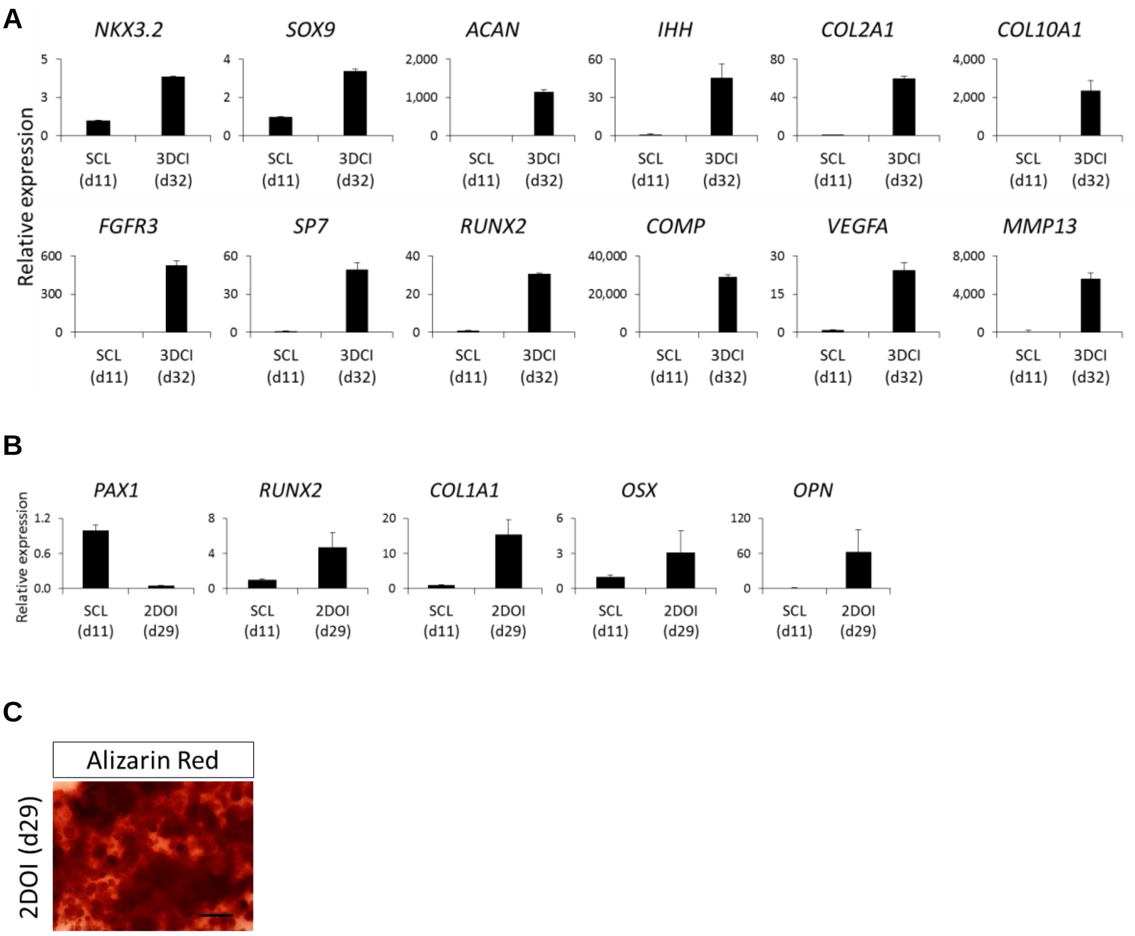

Figure S9. Successful chondrogenesis and osteogenesis from SCL.

(A) RT-qPCR analysis using cartilage markers at day 21 of 3DCI.

(B and C) Differentiation ability of SCL toward osteocytes was assessed by RT-qPCR analysis **(B)** and Alizarin Red staining **(C)** at day 18 of 2DOI. Error bars: mean ± SE (n=3).

Development • Supplementary information

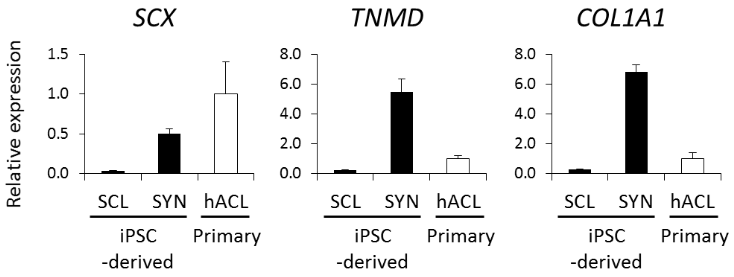

Figure S10. Comparative analysis of iPSC-derived syndetome and primary anterior cruciate ligament cells.

Gene expressions of syndetome-related markers in induced SYN and primary ACL cells were analyzed by RT-qPCR . Error bars: mean ± SE (n=3). ACL, anterior cruciate ligament.

Development • Supplementary information

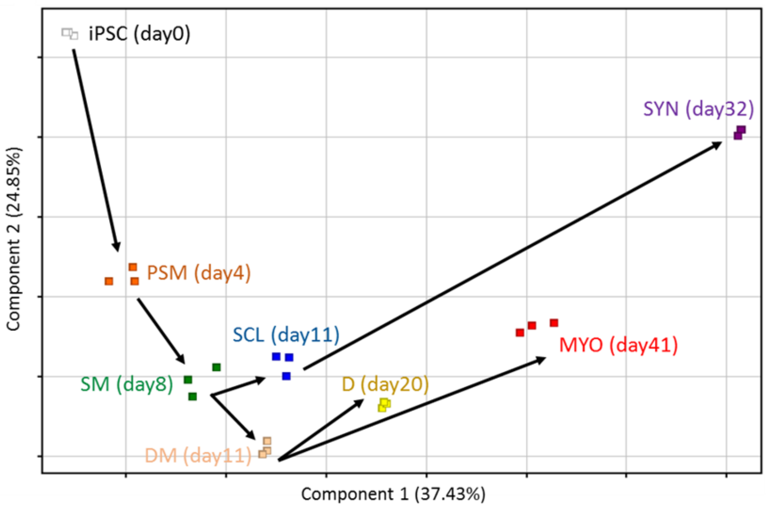

Figure S11. Stepwise induction of four SM derivatives shown by global gene expression analyses.

A PCA plot showing stepwise induction from iPSCs to four derivatives through PSM and SM fates. Three samples were prepared for each lineage.

Development • Supplementary information

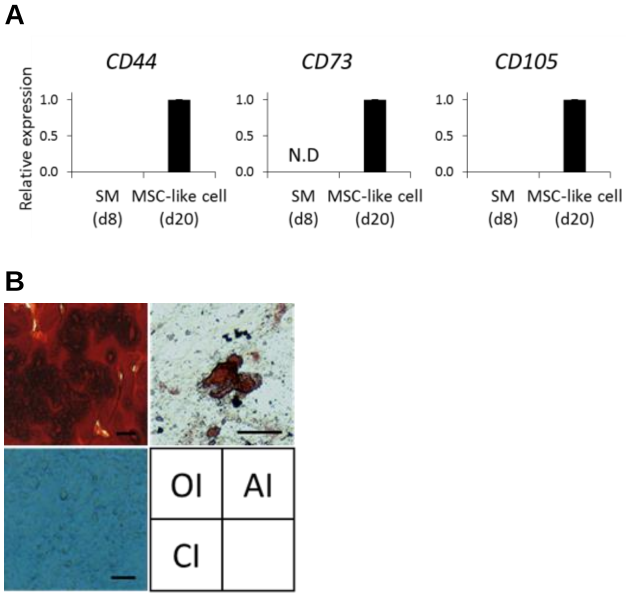

Figure S12. Generation of MSC-like cells from SM.

(A) RT-qPCR analysis of MSC markers at day 12 of the MSC-like cell induction. Error bars: mean ± SE (n=3). N.D, not detected.

(B) Differentiation properties of induced MSC-like cells. The induction of osteogenic, chondrogenic and adipogenic lineages was assessed by Alizarin Red staining (OI), Alcian Blue staining (CI) and Oil Red O staining (AI). Scale bars: 50 μm. OI, osteogenic induction; CI, chondrogenic induction; AI, adipogenic induction.

Development • Supplementary information

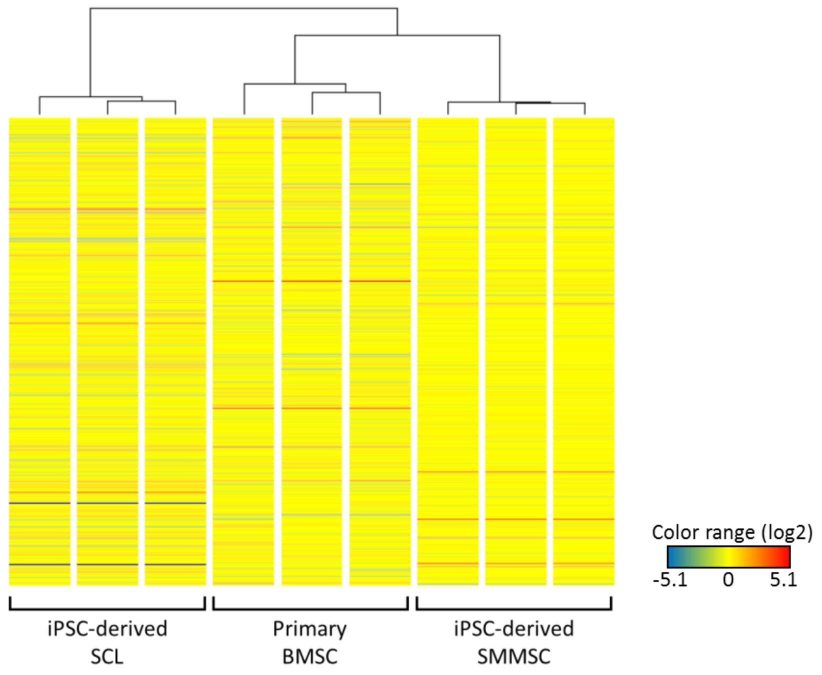

Figure S13. Hierarchical cluster analysis of iPSC-derived SCL, SMMSC-like cell, and primary BMSC.

Genome wide microarray analysis among iPSC-derived SCL, SMMSC-like cells, and primary BMSCs. BMSC, bone marrow mesenchymal stromal cell.

Development • Supplementary information

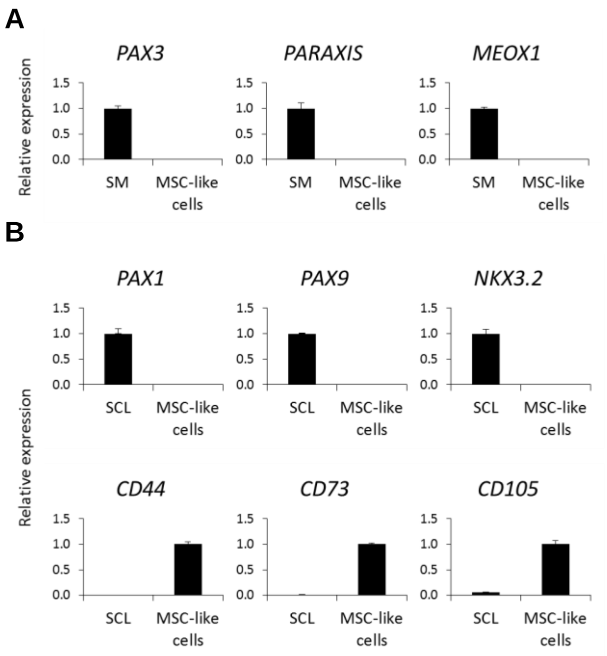

Figure S14. Characterization of induced SM, SCL and MSC-like cells.

(A) Expression comparison of induced SM and MSC-like cells by RT-qPCR analysis.

(B) Expression comparison of induced SCL and MSC-like cells by RT-qPCR analysis.

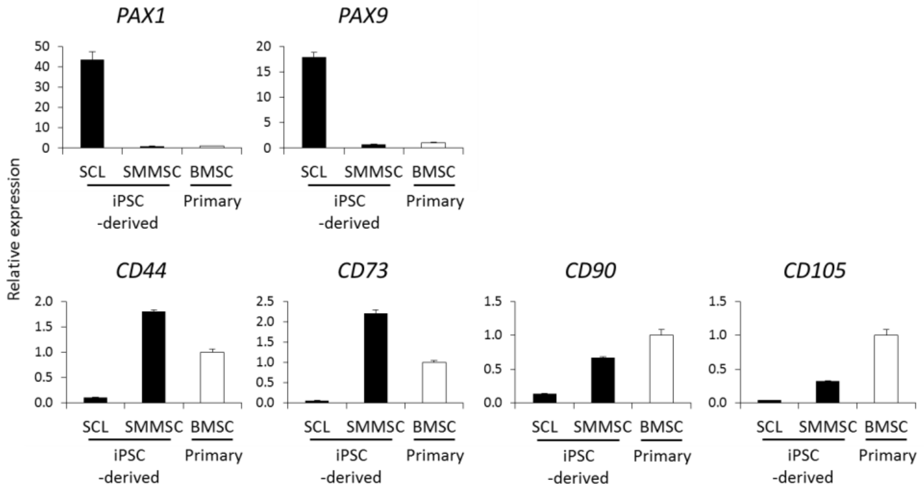

Figure S15. Comparative analysis of iPSC-derived SCL, SMMSC-like cell, and BMSC. Gene expressions of SCL and MSC-related markers in induced SCL, SMMSC-like cells, and primary BMSCs were analyzed by microarray. Error bars: mean ± SE (n=3).

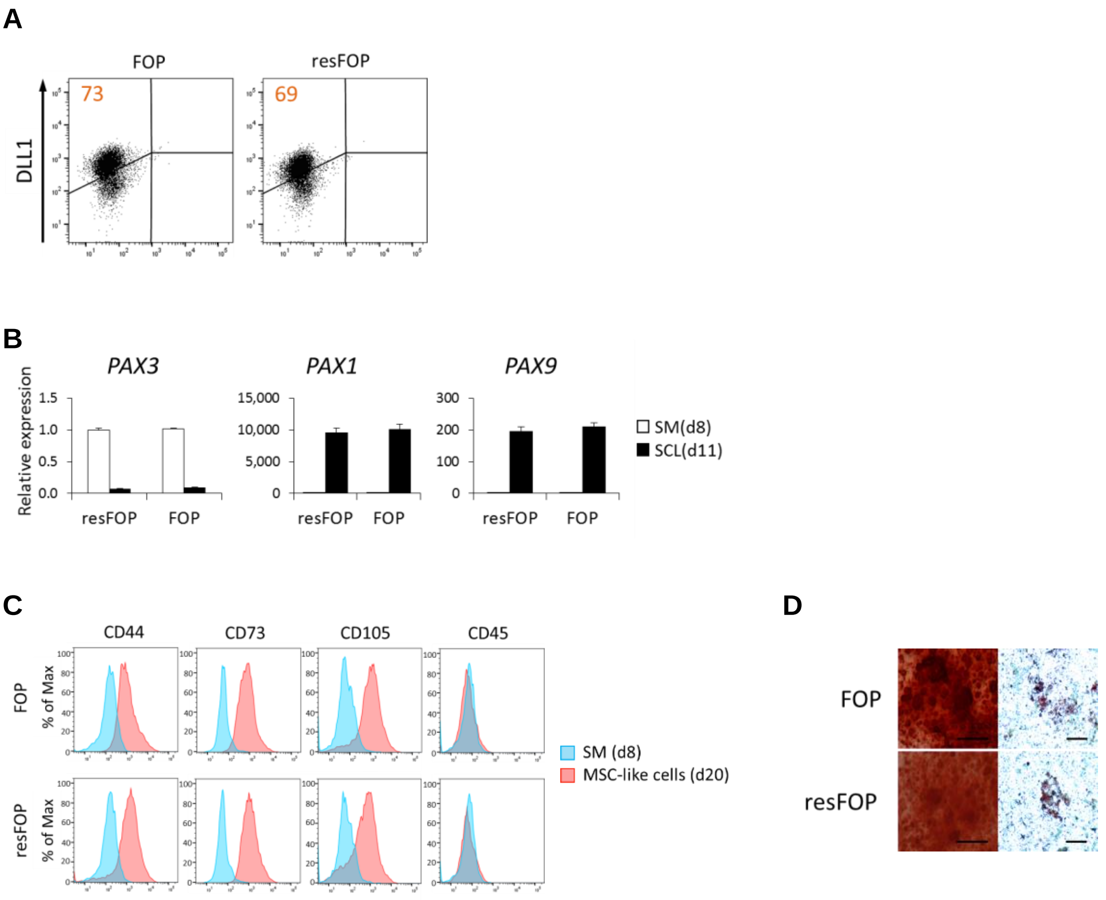

Figure S16. Generation of SCL and MSC-like cells using FOP-iPSCs and resFOP-iPSCs.

(A) $DLL1^+$ cells induced from FOP-iPSCs and resFOP-iPSCs were collected by FACS at day 4 of PSM induction.

(B) Generation of SCL using FOP-iPSCs and resFOP-iPSCs. RT-qPCR analysis using SM and SCL markers at day 3 of SCL induction. Error bars: mean ± SE (n=3).

(C-D) Generation of MSC-like cells using FOP-iPSCs and resFOP-iPSCs. Expressions of surface markers for MSCs were assessed by FACS at day 12 of MSC-like cell induction **(C)**. Differentiation properties of MSC-like cells were assessed by osteogenic and adipogenic induction. These inductions were evaluated by Alizarin Red staining (OI) and Oil Red O staining (AI), respectively **(D)**. Both FOP-MSC-like cells and resFOP-MSC-like cells underwent osteogenic differentiation well, with no difference between them. Scale bars: 50 μm. resFOP, Patient 1 resFOP clone 1; FOP, Patient 1 FOP clone 1.

Development • Supplementary information

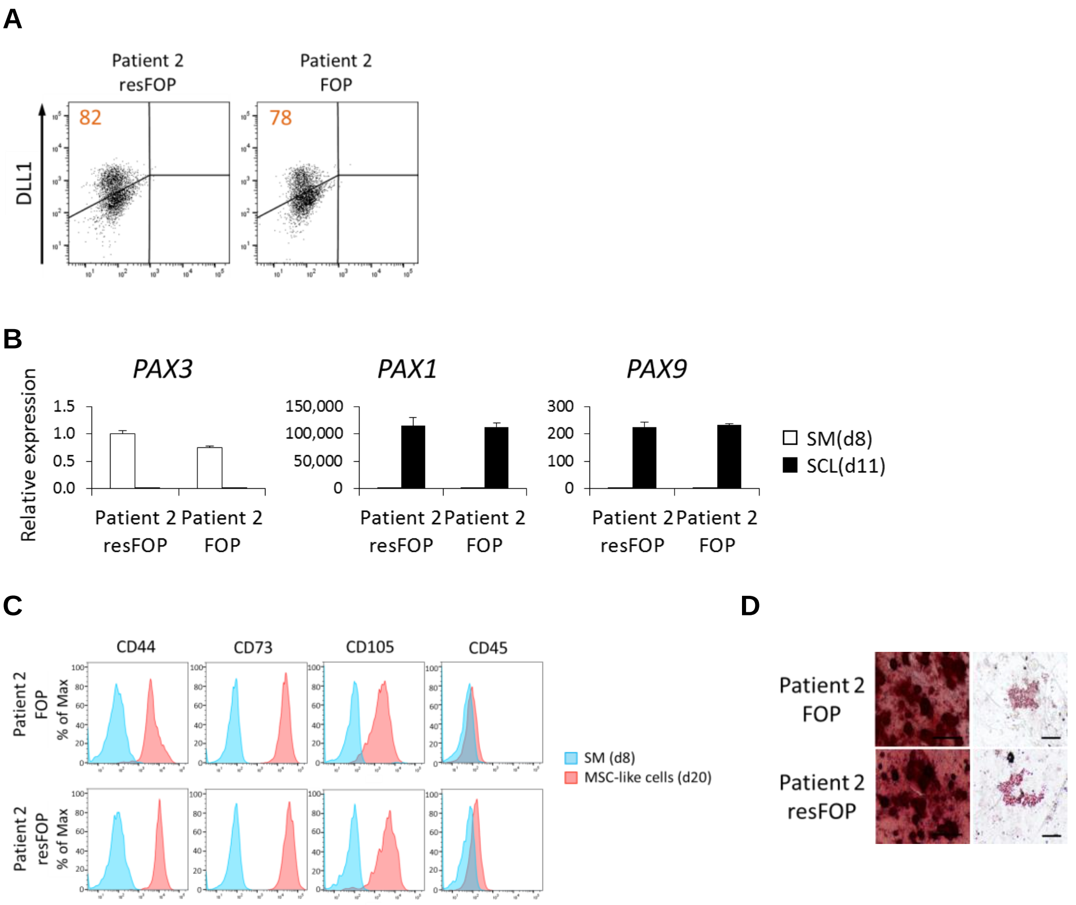

Figure S17. Generation of SCL and MSC-like cells from Patient 2 FOP-iPSCs and resFOP-iPSCs.

(A) DLL1^{+} cells induced from Patient 2 FOP-iPSCs and resFOP-iPSCs were collected by FACS at day 4 of PSM induction.

(B) Generation of SCL derived from Patient 2 FOP-iPSCs and resFOP-iPSCs. RT-qPCR analysis using SM and SCL markers at day 3 of SCL induction. Error bars: mean ± SE (n=3).

(C-D) Generation of MSC-like cells from Patient 2 FOP-iPSCs and resFOP-iPSCs. Expressions of surface markers for MSCs were assessed by FACS at day 12 of MSC-like cell induction **(C)**. Differentiation properties of MSC-like cells were assessed by osteogenic and adipogenic induction. These inductions were evaluated by Alizarin Red staining (OI) and Oil Red O staining (AI), respectively **(D)**. Scale bars: 50 µm; Patient 2 resFOP, Patient 2 resFOP clone 1; Patient 2 FOP, Patient 2 FOP clone 1.

Development • Supplementary information

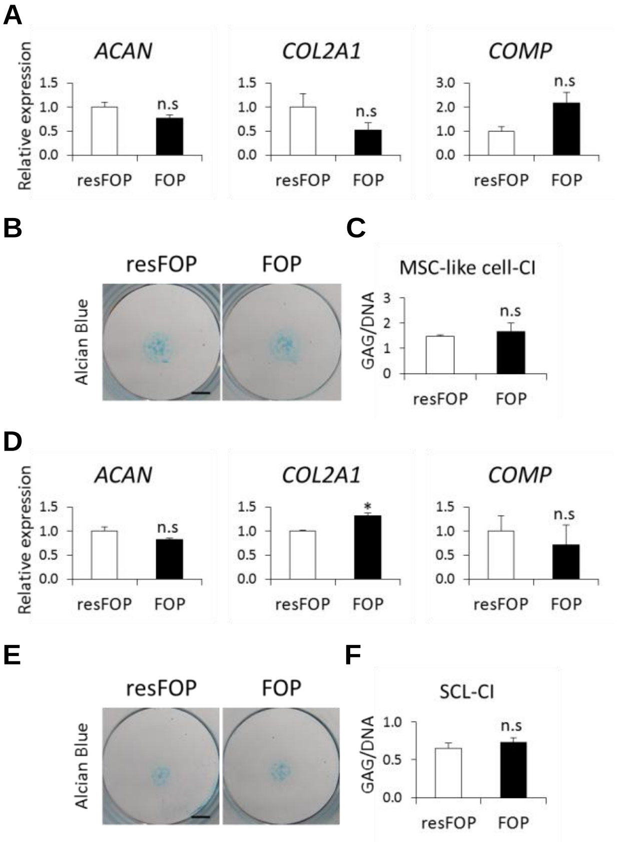

Figure S18. MSC-like cell-CI and SCL-CI derived from FOP-iPSCs and resFOP-iPSCs without Activin A.

(A-C) Evaluation of MSC-like cell-CI derived from FOP-iPSCs and resFOP-iPSCs without Activin A. Chondrogenic differentiation was assessed at day 5 of CI by RT-qPCR analysis **(A)**, Alcian Blue staining **(B)** and GAG/DNA analysis **(C)**.

(D-F) Evaluation of SCL-CI derived from FOP-iPSCs and resFOP-iPSCs without Activin A. Chondrogenic differentiation was assessed at day 5 of CI by RT-qPCR analysis **(D)**, Alcian Blue staining **(E)** and GAG/DNA analysis **(F)**. Error bars: mean ± SE (n=3). *$p < 0.05$ by Student's *t*-test compared with resFOP (A, C, D and F). Scale bars: 200 μm; n.s, no significant difference; resFOP, Patient 1 resFOP clone 1; FOP, Patient 1 FOP clone 1.

Development • Supplementary information

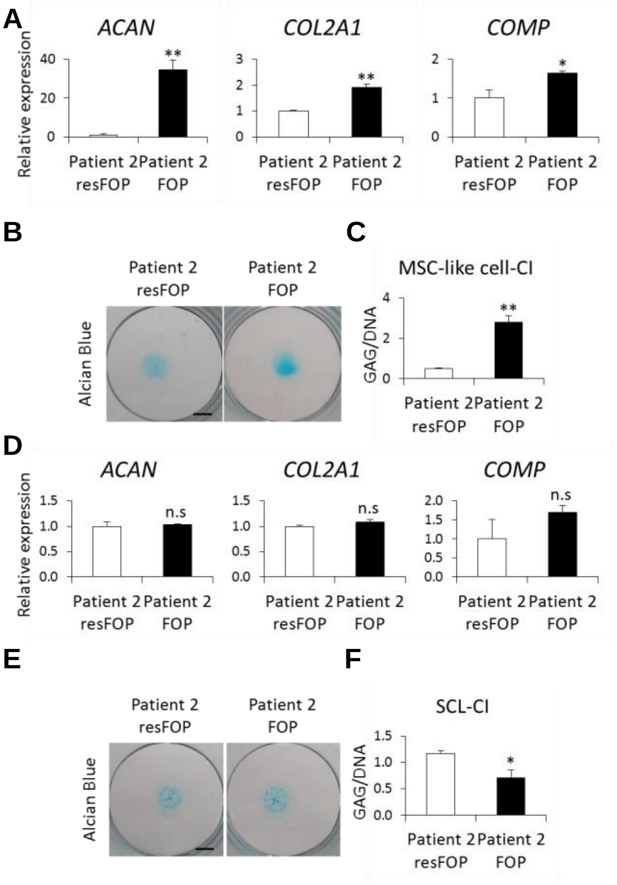

Figure S19. MSC-like cell-CI and SCL-CI derived from Patient 2 FOP-iPSCs and resFOP-iPSCs.

(A-C) Evaluation of MSC-like cell-CI derived from Patient 2 FOP-iPSCs and resFOP-iPSCs. Chondrogenic differentiation was assessed at day 5 of CI by RT-qPCR analysis **(A)**, Alcian Blue staining **(B)** and GAG/DNA analysis **(C)**.

(D-F) Evaluation of SCL-CI derived from Patient 2-FOP-iPSCs and resFOP-iPSCs. Chondrogenic differentiation was assessed at day 5 of CI by RT-qPCR analysis **(D)**, Alcian Blue staining **(E)** and GAG/DNA analysis **(F)**. Error bars: mean ± SE (n=3). *p < 0.05; **p < 0.01 by Student's *t*-test compared with Patient 2 resFOP (A, C, D and F). n.s, no significant difference; Scale bars: 200 µm.

Development • Supplementary information

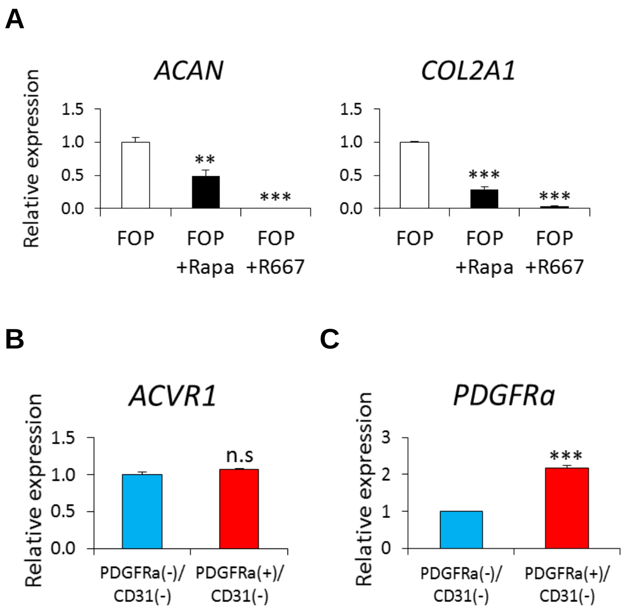

Figure S20. Characterization of FOP-iPSC-derived MSC-like cells and MSC-like cell-CI. **(A)** Evaluation of R667 and Rapamycin treatment on chondrogenesis. Chondrogenic differentiation was assessed by RT-qPCR analysis. Cells were harvested at day 5 of 2DCI and treated with or without R667, Rapamycin.

(B and C) Evaluation of isolated PDGFRα^{+}/CD31^{-} MSC-like cells and PDGFRα^{-}/CD31^{-} MSC-like cells. *ACVR1* **(B)** and *PDGFRa* **(C)** expression were assessed by RT-qPCR analysis. Error bars: mean ± SE (n=3). ***p < 0.001 by Student's *t*-test compared with FOP (A) and PDGFRα^{-}/CD31^{-} population (B and C). Rapa, Rapamycin; n.s, no significant difference.

Development • Supplementary information

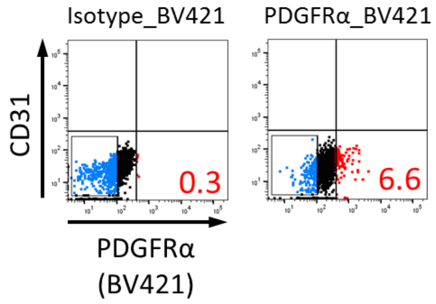

Figure S21. Staining of isotype control antibody for PDGFRa.

FACS analysis of SMMSC-like cells induced from FOP-iPSCs using isotype control antibody for PDGFRa.

Development • Supplementary information

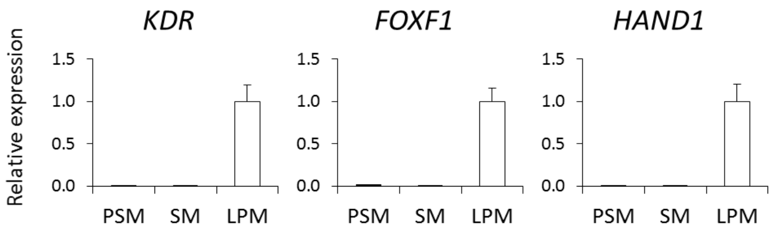

Figure S22. Monitoring contamination of LPM cells at PSM and SM stages.

Contamination of LPM cells in PSM and SM stage was assessed by RT-qPCR using LPM-related markers. iPSC-derived LPM were acquired by the following published protocols (Iyer *et al*., 2015).

Development • Supplementary information

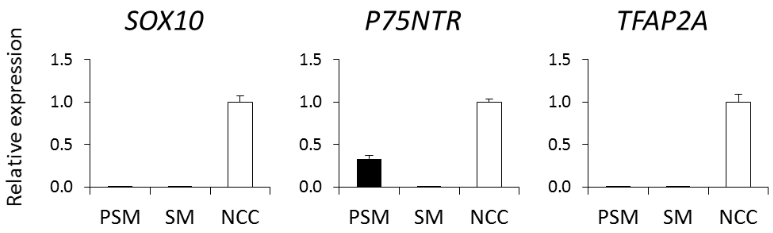

Figure S23. Monitoring contamination of NCCs at PSM and SM stages.

Contamination of NCCs in PSM and SM stages was assessed by RT-qPCR using NCC-related markers. iPSC-derived NCC were acquired by the following published protocols (Fukuta *et al*., 2014).

Development • Supplementary information

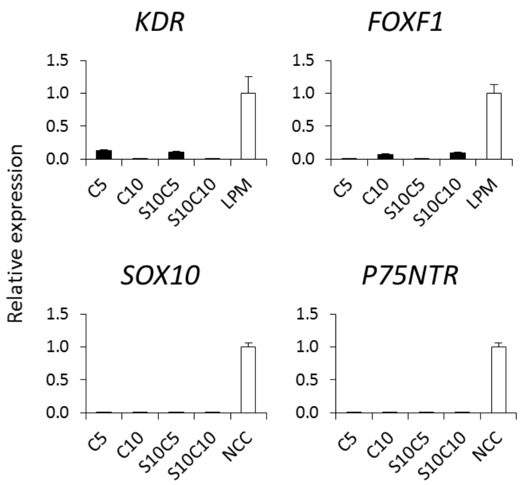

Figure S24. Monitoring contamination of LPM cells and NCCs in SM induced with various conditions.

Contaminations of LPM cells and NCCs were assessed by RT-qPCR. Error bars: mean ± SE (n=3).

Development • Supplementary information

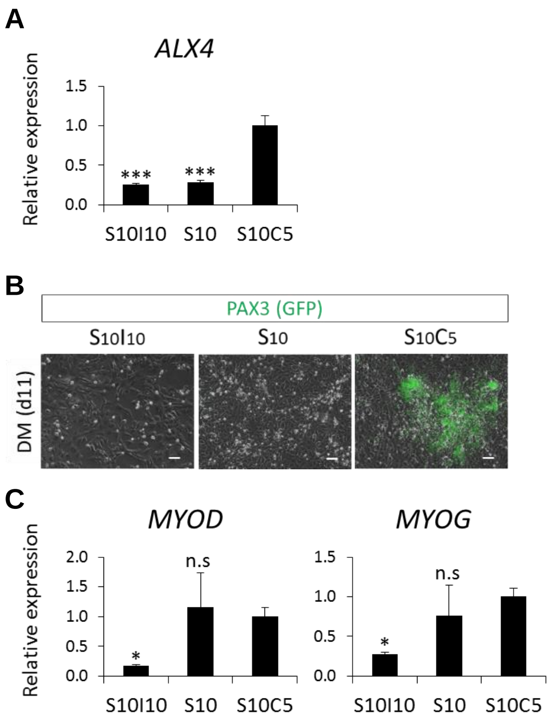

Figure S25. Characterization of DM and MYO differentiated from SM induced with S10I10, S10 or S10C5.

(A-B) Quality of induced DM was assessed by RT-qPCR **(A)** and PAX3-GFP fluorescence **(B)** analyses**.** Scale bars: 50 μm.

(C) Quality of induced MYO was assessed by RT-qPCR analysis. Error bars: mean ± SE (n=3). *** p< 0.001; * p< 0.05 by Dunnett's multiple comparisons *t*-test compared with S10C5 (A, C). n.s, no significant difference.

Development • Supplementary information

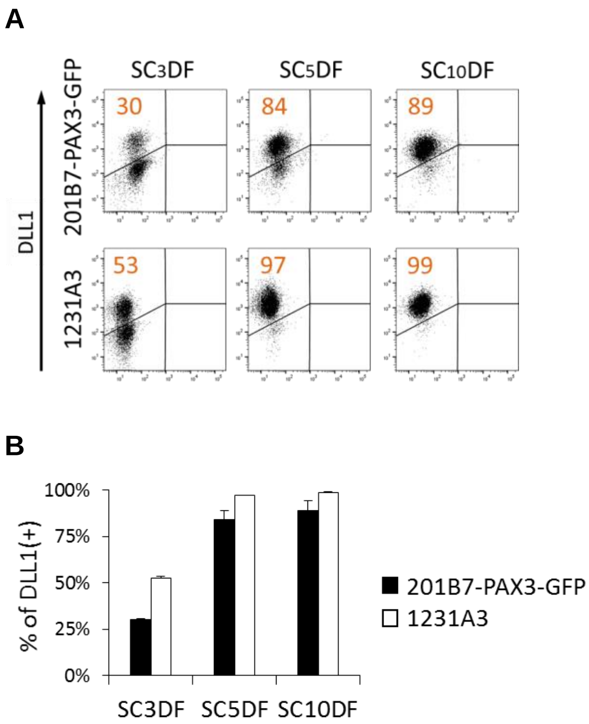

Figure S26. Effect of CHIR99021 concentration during PSM differentiation.

(A-B) FACS analysis at day 4 using anti-DLL1 antibody. iPSC lines, 201B7-PAX3-GFP and 1231A3 were cultured in various CHIR99021 concentrations (SC3DF, SC5DF, SC10DF). Error bars: mean ± SE (n=3). S, SB431542 10 μM; C3, CHIR99021 3 μM; C5, CHIR99021 5 μM; C10, CHIR99021 10 μM; D, DMH1 2 μM; F, FGF2 20 ng/mL.

Development • Supplementary information

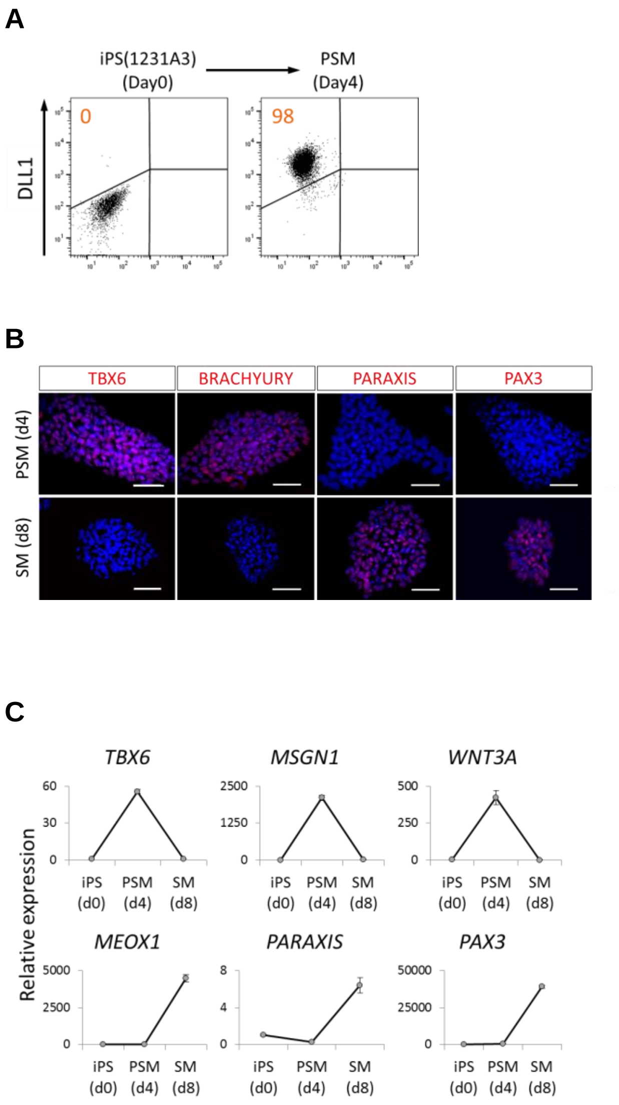

Figure S27. Induction of SM though PSM fate in xeno-free condition.

(A-C) Induction of SM though PSM fate in xeno-free condition. 1231A3, xeno-free and feeder-free iPSCs were used for the experiments. DLL1^{+} cells were collected by FACS at day 4 of induced PSM **(A)**. Differentiation toward SM though PSM fate was assessed by immunocytochemistry **(B)** and RT-qPCR **(C)** analyses. Error bars: mean ± SE (n=3). Scale bars: 50 μm.

Development • Supplementary information

Table S1. Primer sequences for qPCR analysis.

NAME	Forward	Reverse
ACTB	CACCATTGGCAATGAGCGGTTC	AGGTCTTTGCGGATGTCCACGT
NANOG	GGCTCTGTTTTGCTATATCCCCTAA	CATTACGATGCAGCAAATACAAGA
OCT3/4	AGACCATCTGCCGCTTTGAG	GCAAGGGCCGCAGCTT
SOX2	TGGTCCTGCATCATGCTGTAG	AACCAGCGCATGGACAGTTAC
TBX6	AGCCTGTGTCTTTCCATCGT	AGGCTGTCACGGAGATGAAT
MSGN1	GGAGAAGCTCAGGATGAGGA	GTCTGTGAGTTCCCCGATGT
WNT3A	CAAGATTGGCATCCAGGAGT	ATGAGCGTGTCACTGCAAAG
DLL1	TGTGCCTCAAGCACTACCAG	TTCTGTTGCGAGGTCATCAG
BRACHYURY	ACCCAGTTCATAGCGGTGAC	CATTGGGAGTACCCAGGTTG
MEOX1	GAGATTGCGGTAAACCTGGA	GAACTTGGAGAGGCTGTGGA
PARAXIS	TCCTGGAGAGCTGTGAGGAT	CACACCCTGTCACCAACAGT
PAX3	AGGAAGGAGGCAGAGGAAAG	CAGCTGTTCTGCTGTGAAGG
MYOD	CACTCCGGTCCCAAATGTAG	TTCCCTGTAGCACCACACAC
MYOG	TGGGCGTGTAAGGTGTGTAA	CGATGTACTGGATGGCACTG
PAX7	GGGATTCCCTTTGGAAGTGT	CGGCAAAGAATCTTGGAGAC
FGFR4	CTGACCTTCGGACCCTATTCC	GCTGAAGACAGAATCGCTGG
NCAD	AGGATCAACCCCATACACCA	TGGTTTGACCACGGTGACTA
PAX1	CGTCAGCATCCCGCGCTCAT	ACACGCCGTGCTGGTTGGAG
PAX9	ACAGTGCTGCTCCTTCTGGT	ATGTGAGACCTGGGAATTGG
NKX3.2	CAGAAATTCTCCCAAAGATGC	TCTCCCTACAGTTTCGCCG
EN1	GACTCGGACAGGTGCTATCG	AGTTCGCAGTTTCGTCCCTT
ALX4	TCCACTGCATATGAGCTGC	GTTGTTGCCGAGCCAGGA
NOGGIN	CCTCATCGAACACCCAGACC	CATGAAGCCTGGGTCGTAGTG
MSX1	CACACTGCTCCAGTTTCACC	AGGGACTCTTCCAGCCACTT
EGFL6	AAGGCATCACGGGTTGTTAG	CGGTGTATCCTGGAAAGCAT
PDGFRa	GGCCCCATTTACATCATCAC	CATAGCTCCGTGTGCTTTCA
DLK1	CGGCTTCATCGACAAGACCT	GTGAGACCTGTGAACTCGGG
SCX	CCCAAACAGATCTGCACCTTC	GCGAATCGCTGTCTTTCTGTC
MKX	CGCACAGACACTCTGGAAAA	AGCGGCACTTTGACAGTCTT

Development • Supplementary information

COL1A1	GGACACAGAGGTTTCAGTGGT	GCACCATCATTTCCACGAGC
COL1A2	GGATGAGGAGACTGGCAACC	TTGCCCTCAGCAACAAGTTC
CD44	GCAATGCTTCTCAGACCACA	GAGGGGAGAGGGTAGACAGG
CD73	GCCGCTTTAGAGAATGCAAC	CTCGACACTTGGTGCAAAGA
CD105	CACTAGCCAGGTCTCGAAGG	CTGAGGACCAGAAGCACCTC
PAI1	TCCAGCAGCTGAATTCCTG	GCTGGAGACATCTGCATCCT
MMP1	GTGTCTCACAGCTTCCCAGCGAC	GCACTCCACATCTGGGCTGCTTC
ACVR1	GCGGTAATGAGGACCACTGT	CCCTGCTCATAAACCTG
SOX9	GACTTCCGCGACGTGGAC	GTTGGGCGGCAGGTACTG
ACAN	TCGAGGACAGCGAGGCC	TCGAGGGTGTAGCGTGTAGAGA
IHH	CGGTGGACATCACCACATCA	CGTGGGCCTTTGACTCGTAA
COL2A1	GGCAATAGCAGGTTCACGTACA	CGATAACAGTCTTGCCCCACTT
RUNX2	TTACTTACACCCCGCCAGTC	TATGGAGTGCTGCTGGTCTG
COMP	GTCTTGGACACAACCATGCG	CGCAGCTGATGGGTCTCATA
COL10A1	CCCAGCACGCAGAATCCATC	AGTGGGCCTTTTATGCCTGT
FGFR3	AGTACCTGGACCTGTCGGC	CCTCACATTGTTGGGGACCA
SP7	ATCCAGCCCCCTTTACAAGC	TAGCATAGCCTGAGGTGGGT
VEGFA	CAATCGAGACCCTGGTGGAC	TCTCTCCTATGTGCTGGCCT
MMP13	CATGAGTTCGGCCACTCCTT	CCTGGACCATAGAGAGACTGGA
OPN	GCCAGCAACCGAAGTTTTCA	AACCACACTATCACCTCGGC
CD90	AGGGCACCTACACGTGTGCA	GCACTGGAACTTGAGGCTTCC
KDR	GCGATGGCCTCTTCTGTAAG	ACACGACTCCATGTTGGTCA
HAND1	CACTCTTCCACCCTTTTGGA	TCCTGCGTCTGGTTCTCTTT
FOXF1	TCTTTGTGCGAACAACTTGC	AGCGAAGGAAGAGGAGGAAC
SOX10	GAGCTGGACCGCACACCTTGGG	AACGCCCACCTCCTCGGACCTC
TFAP2A	AAGAGTTCACCGACCTGCTG	AGGGCCTCGGTGAGATAGTT
P75NTR	CCGTTGGATTACACGGTCCA	GACAGGGATGAGGTTGTCGG

Table S2. Antibodies for immunostaining and intracellular flow cytometry analysis.

	NAME	Hosts	Company	Cat. No	Dilution
1st	TBX6	Goat	R&D	AF4744	1/50
	CDX2	Mouse	BioGenex	CDX2-88	1/200
	BRACHYURY	Goat	R&D	AF2085	1/200
	PARAXIS	Rabbit	Santacruz	sc-98796	1/50
	MEOX1	Rabbit	abcam	ab75895	1/50
	CDH11	Mouse	Cell Signaling	13577	1/2000
	PAX3	Mouse	DSHB	Clone:C2	1/500
	MHC(clone: MF20)	Mouse	eBioscience	14-6503	1/800
	MHC(clone: H300)	Rabbit	Santacruz	sc-20641	1/200
	MYOD	Rabbit	abcam	ab133627	1/500
	MYOG	Mouse	Santacruz	sc-12732	1/400
	PAX1	Rabbit	abcam	ab95227	1/50
	PAX9	Rabbit	GeneTex	GTX104454	1/50
	NKX3.2	Rabbit	Sigma	HPA027564	1/50
	Type II collagen	Mouse	ThermoScientific	MS-235	1/500
	ALX4	Goat	Santacruz	sc-22066	1/50
	EN1	Rabbit	abcam	ab70993	1/50
	PDGFRa	Goat	R&D	AF307	1/100
	SCX	Rabbit	abcam	ab58655	1/50
	MKX	Rabbit	Atlas antibodies	A83377	1/50
	COL1A1	Rabbit	abcam	ab34710	1/100
	COL1A2	Rabbit	abcam	ab96723	1/100
2nd	Novex Goat anti Mouse IgG(H+L) seondary antibody555		Invitrogen	A21422	1/500
	Novex Goat anti Rabbit IgG(H+L) seondary antibody555		Invitrogen	A21428	1/500
	Novex Donkey anti Goat IgG(H+L) seondary antibody555		Invitrogen	A21432	1/500
	Novex Goat anti Rabbit IgG(H+L) seondary antibody647		Invitrogen	A21245	1/500
	Novex Donkey anti Goat IgG(H+L) seondary antibody647		Invitrogen	A21447	1/500

Development • Supplementary information

Table S3. Antibodies for FACS.

NAME	Host species	Company	Cat. No	Dilution
DLL1 APC-conjugated	Mouse	R&D	FAB1818A	1/200
CD44 PE-conjugated	Mouse	BD	555479	1/50
CD45 APC-conjugated	Mouse	BD	340943	1/50
CD73 PE-conjugated	Mouse	BD	550257	1/50
CD105 APC-conjugated	Mouse	eBioscience	17-1057	1/50
CD140a BV421-conjugated	Mouse	BD	562799	1/100
CD31 Alexa647-conjugated	Mouse	Biolegend	303112	1/50
BV421 Isotype Control	Mouse	BD	562439	1/100
APC Isotype Control	Mouse	BD	565381	1/200

Supplemental references

Fukuta M., Nakai Y., Kirino K., Nakagawa M., Sekiguchi K., Nagata S., Matsumoto Y., Yamamoto T., Umeda K., Heike T., et al. 2014. Derivation of mesenchymal stromal cells from pluripotent stem cells through a neural crest lineage using small molecule compounds with defined media. *PLoS One,* **9,** e112291.

Iyer D., Gambardella L., Bernard W. G., Serrano F., Mascetti V. L., Pedersen R. A., Talasila A. & Sinha S. 2015. Robust derivation of epicardium and its differentiated smooth muscle cell progeny from human pluripotent stem cells. *Development*.
